# Cerebellar glutamatergic system impacts spontaneous motor recovery by regulating *Gria1* expression

**DOI:** 10.1038/s41536-022-00243-6

**Published:** 2022-09-05

**Authors:** Pallavi Asthana, Gajendra Kumar, Lukasz M. Milanowski, Ngan Pan Bennett Au, Siu Chung Chan, Jianpan Huang, Hemin Feng, Kin Ming Kwan, Jufang He, Kannie Wai Yan Chan, Zbigniew K. Wszolek, Chi Him Eddie Ma

**Affiliations:** 1 Department of Neuroscience, City University of Hong Kong, Tat Chee Avenue, Kowloon Tong, Hong Kong SAR; 2 Department of Neurology, Mayo Clinic, Jacksonville, USA; 3 Department of Neurology, Faculty of Health Science, Medical University of Warsaw, Warsaw, Poland; 4 Department of Biomedical Engineering, City University of Hong Kong, Tat Chee Avenue, Kowloon Tong, Hong Kong SAR; 5 School of Life Sciences, Center for Cell and Developmental Biology and State Key Laboratory of Agrobiotechnology, The Chinese University of Hong Kong, Shatin, Hong Kong SAR; 6 Russell H. Morgan Department of Radiology and Radiological Science, Johns Hopkins University School of Medicine, Baltimore, USA

**Keywords:** Spinocerebellar ataxia, Regeneration and repair in the nervous system

## Abstract

Peripheral nerve injury (PNI) often results in spontaneous motor recovery; however, how disrupted cerebellar circuitry affects PNI-associated motor recovery is unknown. Here, we demonstrated disrupted cerebellar circuitry and poor motor recovery in ataxia mice after PNI. This effect was mimicked by deep cerebellar nuclei (DCN) lesion, but not by damaging non-motor area hippocampus. By restoring cerebellar circuitry through DCN stimulation, and reversal of neurotransmitter imbalance using baclofen, ataxia mice achieve full motor recovery after PNI. Mechanistically, elevated glutamate-glutamine level was detected in DCN of ataxia mice by magnetic resonance spectroscopy. Transcriptomic study revealed that *Gria1*, an ionotropic glutamate receptor, was upregulated in DCN of control mice but failed to be upregulated in ataxia mice after sciatic nerve crush. AAV-mediated overexpression of *Gria1* in DCN rescued motor deficits of ataxia mice after PNI. Finally, we found a correlative decrease in human GRIA1 mRNA expression in the cerebellum of patients with ataxia-telangiectasia and spinocerebellar ataxia type 6 patient iPSC-derived Purkinje cells, pointing to the clinical relevance of glutamatergic system. By conducting a large-scale analysis of 9,655,320 patients with ataxia, they failed to recover from carpal tunnel decompression surgery and tibial neuropathy, while aged-match non-ataxia patients fully recovered. Our results provide insight into cerebellar disorders and motor deficits after PNI.

## Introduction

Injury to the peripheral nervous system (PNS) occurs when a nerve is crushed, transected or the proper communication between the PNS and the central nervous system (CNS) is disrupted (i.e. peripheral neuropathy)^1–7^. Damage to the peripheral nerve is followed by a slow rate of axon regeneration (1–2 mm/day) and is usually associated with a sub-optimal function recovery^1,2,7^. For instance, proximal nerve injuries that involve partial or complete transection of peripheral nerves require long-distance axon regeneration to reinnervate their target muscles, generally resulting in the unsatisfactory recovery of motor function^8^ after prolonged muscle denervation^1,7^. The success of function recovery depends on the reinnervation of denervated target muscle by regenerating axons and collateral sprouting of uninjured axons, and the reorganization of the nervous system circuitry. Over the past decade, there are no significant nerve repair therapeutic advances have been made to improve function recovery, especially for severe nerve injuries. Therefore, immediate attention is required for strengthening our understanding on the causal relationship between the remodeling of central networks and function recovery after PNIs^9–15^.

The cytoarchitecture and neural circuitry system of the cerebellum are highly organized and uniform. Cerebellum integrates the motor and sensory inputs from the PNS to produce a feedback output to the descending motor pathways that control movements. This motor output is precisely modulated by the inhibitory input to the deep cerebellar nuclei (DCN) from Purkinje cells, and the inhibitory Purkinje cell output results in a reduction of excitatory output from DCN to the motor cortex that leads to modification of motor control^16,17^. Dysfunction of Purkinje cells causes ataxia, a neurological disease with clinical features of tremors and loss of motor coordination^18–20^. Clinical and animal studies showed that changes in the somatosensory cortex activities detected by functional magnetic resonance imaging (MRI) were observed following amputation^9^, spinal cord injury^9,10^, and PNI even 1 year after nerve transection and surgical repair^11^. These cortical changes are generally attributed to the basis of post-injury sensory and motor function deficits. Cortical plasticity (cortical map changes) can be either adaptive—compensating the reduction of peripheral sensory input^21^ or maladaptive—potentiating neuropathic pain (i.e. phantom limb pain) and dystonia^22^. Although peripheral nerves could regenerate after lesions^1,2,7^, little is known about how exactly cortical plasticity contributes to the adaptive (promoting effect on growth and survival) or maladaptive responses (failure of axon regeneration, neuropathic pain, and cell death) in the PNS after nerve injuries^1,2,7^.

To address these key questions, we manipulated and directly observed interactions between cerebellar circuitry integrity and functional recovery after PNIs using a Purkinje cell-specific LIM-homeodomain transcription factors *Lhx1* and *Lhx5* conditional double knockout (ataxia) mouse with thinner Purkinje cell dendrites and abnormal dendritic spines (arborization)^23^. By using a combination of animal behavioral, electrophysiological, pharmacological, and MRI approaches, we reveal that cerebellar circuitry disruption and neurotransmitter imbalance delay spontaneous motor recovery after PNIs. Correction of defective cerebellar circuitry by electrical stimulation of DCN or by pharmacological neuromodulation recovered motor function after PNIs. Furthermore, magnetic resonance spectroscopy (MRS) study revealed the imbalance of glutamate-glutamine (Glx) level in the cerebellum of ataxia mice, suggesting the involvement of glutamate receptors. Transcriptomic analysis of ataxic cerebellum revealed the involvement of an α-amino-3-hydroxy-5-methyl-4-isoxazolepropionic acid (AMPA) subtype of ionotropic glutamate receptor Gria1 in spontaneous motor recovery after PNI. In AMAP receptors, there are four subunits, including GluA1–4 (or GluR1–4), which form functionally different tetramers^24^. GluA1 gene deletion impaired short-term spatial memory and enhanced long-term spatial memory, and these mice have been reported to show normal overall locomotor activity^25–27^. *Gria1*, the gene encoding AMPA receptor subunit glutamate A1 (GLUA1), was originally identified as a putative risk gene for schizophrenia. Decreased GRIA1 expression has been found in the hippocampus of schizophrenia patients in three independent studies^28–30^. GluA1 receptor and GluA1 knockout mice have been studied extensively in many aspects of neurological diseases and have been implicated in long-term potentiation, Alzheimer’s disease, schizophrenia, depression, sleep and wakefulness, epilepsy, and neuropathic pain^26,31–36^. However, the roles of cerebellar Gria1 in spontaneous motor recovery after PNI is largely unknown. To address the question of whether Gria1 acts as a key gene for cortical plasticity and PNS regeneration, we showed that intracerebroventricular administration of an AMPA receptor blocker, 6,7-dinitroquinoxaline-2,3-dione (DNQX), impaired functional recovery mimicking the inhibitory effect of ataxia mice on motor recovery following PNI. *Gria1* overexpression via intracerebroventricular delivery of adeno-associated virus (AAV) rescued motor deficits in ataxia mice. Finally, we extended these findings to clinical studies of human GRIA1 expression and motor recovery in patients with ataxic manifestations. Downregulation of human GRIA1 expression in patients with ataxic manifestations was observed across several human Gene Expression Omnibus (GEO) datasets. Our clinical data showed that ataxia patients were unable to recover motor function after carpal tunnel decompression and tibial nerve neuropathy, while non-ataxia patients showed complete motor recovery. These findings reveal cerebellar control of motor recovery that provides the basis for the unexplored link between other cerebellar disorders and poor clinical outcomes following PNIs.

## Results

### Purkinje cell dysfunction affects motor recovery

**Fig. 1:**
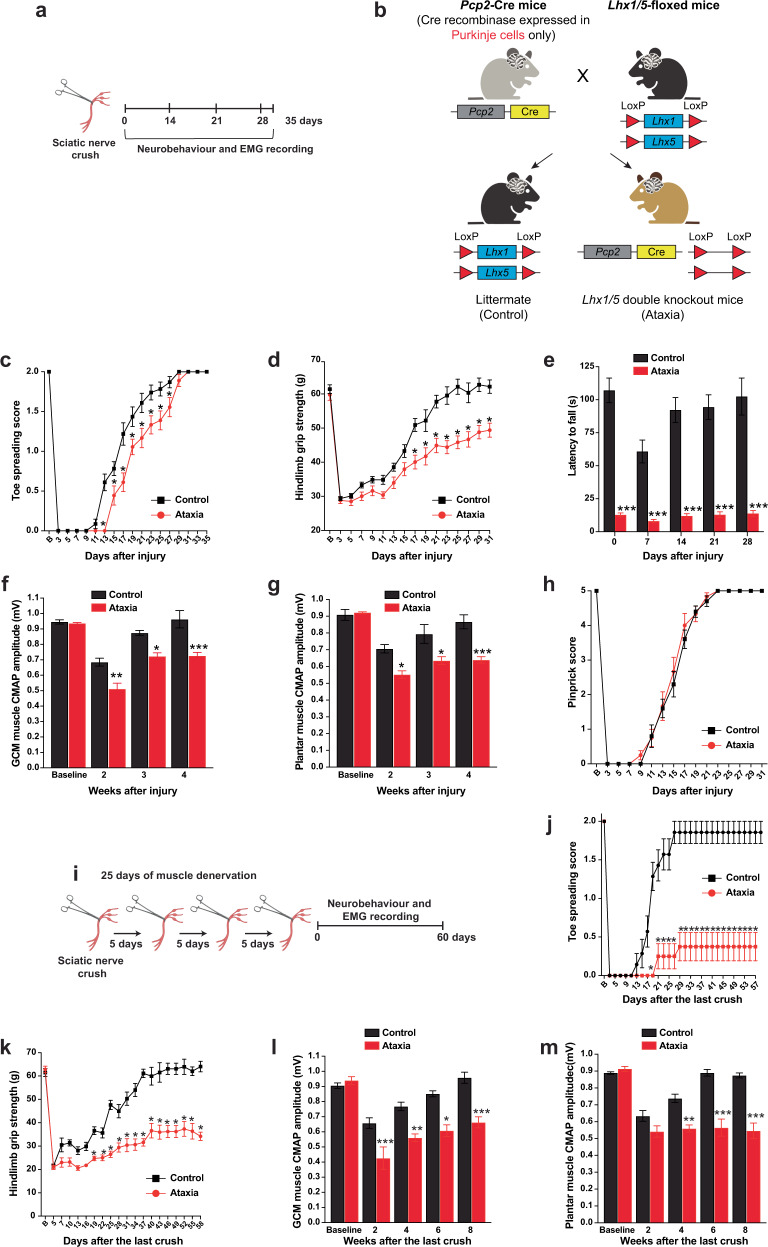
Purkinje cell dysfunction affects motor recovery after sciatic nerve crush (SNC). **a** Schematic diagram illustrating the experimental paradigm for the animal model of SNC. **b** A schematic representation of breeding scheme to generate *Lhx1/5* double knockout mice (ataxia mice). **c** Ataxia mice exhibited delayed toe spreading reflex. **d** Control mice regained full hindlimb grip strength within a month, while ataxia mice recovered 82.6% of grip strength. **e** Ataxia mice displayed shorter latency to fall than control mice throughout the entire testing period. **f** and **g** Functional neuromuscular junction reinnervation was measured by weekly electromyography (EMG) recording of proximal (gastrocnemius, GCM) and distal (interosseous) plantar muscles. **h** No differences between the two mouse groups were detected for sensory function recovery by pinprick assay. **i** Schematic diagram illustrating the experimental paradigm for an animal model of prolonged muscle denervation of 25 days. **j** Ataxia mice only recovered minimal toe spreading reflex within the critical period of muscle denervation in two months, while control mice returned to baselines within one month. **k** The hindlimb grip strength of ataxia mice was the lowest across different muscle denervation models (16 and 22, and 25 days) at 2-month post-SNC. **l** and **m** The compound muscle action potential (CMAP) amplitudes of GCM and plantar muscles in control mice returned to baselines by 4–6 weeks post-SNC although the CMAP values of ataxia mice remained significantly below the baseline values. *n* = 9–10 mice per group for all neurobehavioral experiments; *n* = 6–8 mice per group for EMG recording. Values represent the mean ± SEM; two-way ANOVA followed by post hoc Bonferroni test. *P < 0.05; **P < 0.01; ***P < 0.001 when compared with the control group. Detailed statistics analyses comparing CMAP amplitude data points across time within groups are available in the Supplementary Table 2.

We first determined whether a relationship exists between cerebellar circuitry integrity and spontaneous functional recovery after a single sciatic nerve crush (SNC) (Fig. 1a). We generated the *Lhx1/5* double knockout mice (ataxia mice) by crossing *Purkinje cell protein* (*Pcp*)*2*-*Cre* mice with *Lhx1* floxed (fl) and *Lhx5* fl mice, to obtain adult littermates (*Pcp2*-*Cre*^−/−^*/Lhx1*^fl/fl^/*Lhx5*^fl/fl^) and ataxia mice (*Pcp2*-*Cre*^+/−^/*Lhx1*^fl/fl^/*Lhx5*^fl/fl^) for subsequent experiments^23^ (Fig. 1b). Ataxia mice exhibited delayed toe spreading reflex (Fig. 1c) and was only able to restore 82.6% of hindlimb grip strength, while control mice regained full hindlimb grip strength within a month (Fig. 1d). As expected, poor motor coordination was detected in ataxia mice by accelerated rotarod test. The latency to fall was much shorter in the ataxia mice than in the control group throughout the entire testing period (Fig. 1e). Motor function recovery was further validated by electromyography (EMG) recording^6,7,37^ showing decreased compound muscle action potential amplitude (CMAP) in proximal (gastrocnemius) and distal (interosseous) plantar muscles of ataxia mice at weeks 2, 3 and 4 after injury. The EMG activities of ataxia mice dropped to 77.7% (gastrocnemius) and 69.5% (interosseous) of the baseline values, and the controls were fully recovered in both proximal (Fig. 1f) and distal muscles (Fig. 1g) in about 1 month. The rate of in vivo axon regeneration of sensory axons was not affected in ataxia mice as measured by pinprick assay (Fig. 1h) and sciatic nerve pinch test (Supplementary Fig. 1). This is consistent with our in vitro studies that the intrinsic growth capacity of axotomized and preconditioned (induced maximal growth capacity) sensory dorsal root ganglion (DRG) neurons were not affected in ataxia mice (Supplementary Fig. 2), indicating that the delay in function recovery is motor-specific. Pre-SNC baseline values of motor functions including toe spreading reflex, grip strength and EMG activities were comparable between ataxia and control mice.

Patients with proximal PNI such as cubital tunnel syndrome that requires long-distance axon regeneration often results in unsatisfactory motor function recovery, which depends on the time period between onset of symptoms and surgery termed “critical period”. In mice, motor function recovery is non-existence if regenerating axons arrive at the motor endplate after 37 days (critical period) and regain full motor function recovery only if regenerating axons reach distal muscle within 37 days^1,7^. We have developed a mouse model to study critical periods by performing repeated SNCs to delay regenerating axons from reaching distal muscle for a specific period of time^1,7^. We start with 16 days of muscle denervation by crushing the sciatic nerve every 2 days as it takes 10 days for the regenerating axons to reach the distal target muscle^1^ (Supplementary Fig. 3a). We found that there was an 8 days delay in reaching full toe spreading reflex (Supplementary Fig. 3b) as well as a partial restoration of grip strength (75.6%) (Supplementary Fig. 3c) and reduction of EMG activities (68.1–71.8%) in ataxia mice (Supplementary Fig. 3d, e). Notably, ataxia mice were not able to recover toe spreading reflex when we increased the total distance of axon regrowth by extending the period of muscle denervation to 22 days (within the critical period) (Supplementary Fig. 4a), while the toe spreading reflex of control mice returned to baseline levels at days 31 (Supplementary Fig. 4b). Both 16-day and 22-day muscle denervation ataxia mouse groups showed a similar reduction of grip strength (Supplementary Fig. 4c) and EMG activities (Supplementary Fig. 4d, e) while the control mice were fully recovered approximately 1 month after the last crush. Extending the time period of muscle denervation to 25 days (Fig. 1i), ataxia mice only restored 18.7% of toe spreading reflex (Fig. 1j) and exhibited the lowest grip strength (Fig. 1k) and CMAP values 2 months after the last crush (Fig. 1l, m) when compared with 16- and 22-days muscle denervation ataxia mouse groups. The sensory function recovery remained unaffected across different muscle denervation models (16, 22, and 25 days) in both control and ataxia mice (Supplementary Fig. 5a). However, motor coordination of ataxia mice further dropped to 10.8–34.7% of the pre-SNC baseline (15.4–21.1 s) values 2 months after 16-, 22- and 25-days of muscle denervation. Ataxia mice were not able to stay on the rotarod for more than 7 s after SNC (Supplementary Fig. 5b). It provides further evidence that Purkinje cell dysfunction results in irreversible motor deficits after a single SNC and shorten the critical period to 22 days that usually show full motor function recovery in control mice. The severity of motor deficit is correlated directly with the time period of muscle denervation in ataxia mice.

### Cerebellar circuitry integrity determines motor recovery

**Fig. 2:**
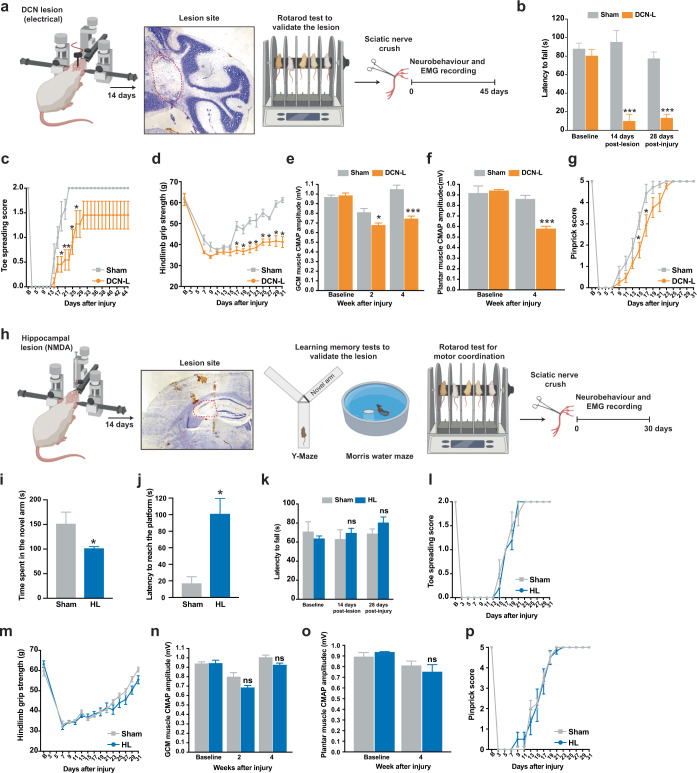
Cerebellar circuitry integrity determines motor recovery. **a** Schematic diagram illustrating the experimental paradigm for an animal model of deep cerebellar nuclei lesion (DCN-L). Electrical DCN-L was performed and followed by a single sciatic nerve crush (SNC). Site of DCN-L (red dotted line) was confirmed by cresyl violet staining. **b** Motor coordination was severely impaired in DCN-L mice as assessed by an accelerated rotarod test. **c** DCN-L mice were unable to fully recover toe spreading reflex. **d** The hindlimb grip strength of DCN-L mice was remained significantly below the baseline values after SNC. **e** and **f** The compound muscle action potential (CMAP) amplitude of DCN-L mice was significantly lower than the sham-operated mice at 4-week post-SNC. **g** A slight delay in sensory function recovery was observed in DCN-L mice before they returned to baseline levels on day 25. **h** Schematic diagram illustrating the experimental paradigm for an animal model of the hippocampal lesion (HL) by microinjection of N-methyl-d-aspartate. The lesion site (red dotted line) was confirmed by cresyl violet staining. Learning and memory impairment were detected by Y-Maze and Morris water maze, followed by SNC. **i** HL mice spent less time exploring the novel arm indicating the impairment of spatial recognition memory after the hippocampal lesion. **j** HL mice spent more time finding the hidden platform than the control mice during the Morris water maze probe test after 5 training days. **k** Motor coordination was not affected in hippocampus-lesioned mice before and after the SNC as assessed by the accelerated rotarod test. **l**–**p** No major differences were detected between HL and sham mice in motor and sensory function baselines. Both mouse groups returned to pre-SNC baseline values about one month after SNC. *n* = 7–8 mice per group (sham and DCN-L). *n* = 5-7 mice per group (sham and HL). Values represent the mean ± SEM; two-way ANOVA followed by post hoc Bonferroni test except in **i** and **j**, unpaired Student’s *t*-tests were used; *P < 0.05; ***P < 0.001 when compared with the sham-operated mice in DCN-L or HL. ns non-significant. Detailed statistics analyses comparing CMAP amplitude data points across time within groups are available in Supplementary Table 2.

Peripheral deafferentation or limb amputation induces massive plasticity changes within cortical and sub-cortical motor areas^38^, however; there is limited understanding of how the cerebellar circuitry affects motor recovery after PNS injury. We, therefore, examine the role of DCN in motor recovery after PNI by electrical DCN lesions or to lesion a non-motor area such as hippocampus as control. Successful DCN lesion was confirmed by motor coordination deficit assessed by accelerated rotarod test and histological analysis, and only those DCN-lesioned mice with ataxia symptom were used for SNC study (Fig. 2a). DCN-lesioned mice showed 87.5% reduction of latency to fall from the accelerated rotarod (Fig. 2b). Consistent with the single SNC study on ataxia mice, DCN-lesioned mice showed delayed in motor function recovery by regaining 72.5% of toe spreading reflex 44 days after SNC, whereas sham-operated mice reached full recovery of toe spreading reflex by day 23 post-SNC (Fig. 2c). DCN-lesioned mice were unable to recover hindlimb grip strength back to the average pre-SNC baseline value of 61.5 g and remained significantly below the baseline values from day 7 onwards (36.3−41.2 g) (Fig. 2d). The CMAP amplitude of DCN-lesioned mice were significantly lower than the sham-operated mice in both the gastrocnemius muscle (70.9% of sham-operated) (Fig. 2e) and interosseous muscles (67.3% of sham-operated) at 4-week post-SNC (Fig. 2f). A slight delay in sensory function recovery was observed at days 15 and 17 in DCN-lesioned mice before they returned to baseline levels at day 25, which was 2 days after the sham-operated mice reaching full sensory function after SNC (Fig. 2g). DCN lesion did not affect the motor (toe spreading reflex, grip strength and EMG) and sensory (pinprick) baseline values, when compared with the sham-operated mice.

To examine if alternation of non-motor circuitry would have any impacts on motor recovery after PNI, a hippocampal lesion was performed since hippocampus has long been considered the major brain region for learning and memory. Damage to the hippocampus reduces learning ability and causes severe memory loss in humans without affecting locomotor activities^39–41^. In mice, surgical manipulation in hippocampus did not affect motor activities, assessed by accelerating rotarod and open field test^42^. We, therefore, performed cytotoxic hippocampal lesion by microinjection of N-methyl-d-aspartate (NMDA), which has been shown to produce a selective removal of the hippocampus and dentate gyrus, resulting in severe spatial cognitive deficits^42–44^. Microinjection of NMDA was performed 3 weeks before the SNC, and learning and memory deficits were validated in hippocampus-lesioned mice by Y-maze and Morris water maze^43^, and histological analysis (Fig. 2h). The sham-operated mice (151.9 ± 23.2 s) spent more time in exploring the novel arm than the hippocampus-lesioned mice (102 ± 2.9 s) indicating the impairment of spatial recognition memory after the hippocampal lesion (Fig. 2i). Performance in the Morris water maze was impaired in the hippocampus-lesioned mice compared to sham-operated mice. The time to reach the platform (latency) was reduced significantly in sham-operated mice (17.3 ± 7.6 s) during the probe test after 5 training days, while the hippocampus-lesioned mice (100 ± 19.4 s) spent more time finding the hidden platform (Fig. 2j). Motor coordination was not affected in hippocampus-lesioned mice before and after the SNC as assessed by accelerated rotarod test (Fig. 2k). There were no major differences between hippocampus-lesioned and sham-operated mice in motor and sensory baseline values. The hippocampal lesion did not affect motor and sensory functional recovery, and EMG activities since all the mice recovered to pre-SNC baseline values in about one month after a single SNC (Fig. 2l–p).

### Integrity of descending motor circuits in ataxia

**Fig. 3:**
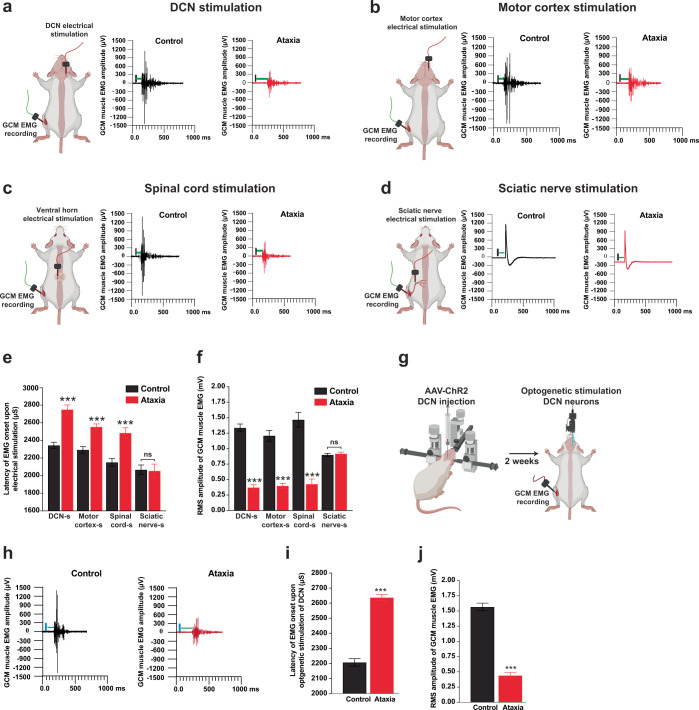
Impairment of cerebellothalamocortical motor circuitry in ataxia mice. **a**–**d** Electromyography (EMG) responses evoked by deep cerebellar nuclei (DCN), motor cortex, spinal cord and sciatic nerve stimulations were recorded from gastrocnemius (GCM) muscle in anesthetized mice. The vertical black line marks the time of electrical stimulations and the horizontal green line represents the latency of EMG onset. **e** Ataxia mice showed significant increase in latency of EMG onset. **f** Spontaneous EMG activities reduced significantly in ataxia mice with DCN, motor cortex, and spinal cord stimulations. By contrast, the compound muscle action potential (CMAP) amplitudes evoked by sciatic nerve stimulation, remained unchanged and were comparable between ataxia and control mice. **g** and **h** EMG activities of GCM muscle upon optogenetic stimulation of AAV2-mediated channelrhodopsin-2 (ChR2)-transduced DCN neurons. **i** and **j** Similar to the studies on electrical stimulation of DCN, ataxia mice showed a significantly longer latency of EMG onset than control mice and a reduction of CMAP amplitude upon optogenetic stimulation of DCN. *n* = 6–8 mice per group were used in electrical and optogenetic stimulations. About 25–30 EMG responses per mouse were used for CMAP amplitudes and latency analysis. Values represent the mean ± SEM; ***P < 0.001, unpaired Student’s *t*-test. ns non-significant.

Execution of motor function depends on the intricate integration of descending motor pathways that ultimately activate target muscles. Muscle contractions can be readily evoked by electrical stimulation of motor-related brain areas and multiple sites along the descending motor circuitry^45–47^. To assay the cerebellothalamocortical motor circuitry, we applied electrical stimulation over the DCN, motor cortex and ventral horn of spinal cord (L2–L5) followed by CMAP amplitude recording in the hindlimb gastrocnemius muscle (Fig. 3a–c). There was a delay of 261–407 ms between neural firing and EMG onset in ataxia mice when we stimulated the DCN, motor cortex and spinal cord, compared to control mice. However, the nerve transduction velocity of sciatic nerve was not affected in ataxia mice, indicating that the infrastructure (i.e. myelination) of the sciatic nerve remained unaffected (Fig. 3d, e). Spontaneous EMG activities reduced significantly in ataxia mice with DCN, motor cortex and spinal cord stimulations. By contrast, the CMAP amplitudes evoked by sciatic nerve stimulation, remained unchanged and were comparable between ataxia and control mice (Fig. 3f). Contrarily to electrical stimulation whereas current flow to neighboring structure occurs, optogenetics has the great advantage not to interfere with the neighboring tissues. We recorded EMG activities of gastrocnemius muscle upon optogenetic stimulation of AAV2-mediated channelrhodopsin-2 (ChR2)-transduced DCN neurons^48^ (Fig. 3g, h) with high efficiency (Supplementary Fig. 6). Ataxia mice showed a significant longer latency of EMG onset than control mice (Fig. 3i) and reduction of CMAP amplitude following optogenetic stimulation of DCN with similar trends observed in DCN electrical stimulation (Fig. 3j). Our findings suggest that changes in cerebellar outputs in ataxia mice alters spinal motor circuits in the descending motor pathway, results in reduced motor recovery after SNC injury.

### Restoration of cerebellar circuitry and improvement of DCN hyperexcitability recover motor function recovery

Cerebellar deep brain stimulation (DBS) (i.e. interposed nucleus of DCN) and pharmacologic intervention (i.e. baclofen) have been shown to improve ataxia symptoms by restoring aberrant hyperexcitability in the cerebellum^49,50^. We, therefore, argue that restoration of cerebellar circuitry by cerebellar DBS or baclofen immediately after SNC could recover motor function in ataxia mice. First, we recorded neural firing in the interposed nucleus of DCN in freely moving mice and observed intense activity spikes with aberrant and irregular waveforms indicating abnormal cerebellar circuitry in ataxia mice (Supplementary Fig. 7a). Sorted DCN neuronal spikes showed that there were significant increases in mean neural firing rate (Supplementary Fig. 7b) and a number of bursts (Supplementary Fig. 7c), and a decrease in the interspike interval (Supplementary Fig. 7d, e) in DCN neurons of ataxia mice compared to control mice. Interestingly, there was a trend of increase in neural firing and number of bursts in control mice at 2-week post-SNC onward, and the increment was more prominent in ataxia mice. It is possible that deafferentation due to SNC results in neuroplasticity modification in the cerebellum since cortical reorganization occurs following sciatic nerve injury^13^.

**Fig. 4:**
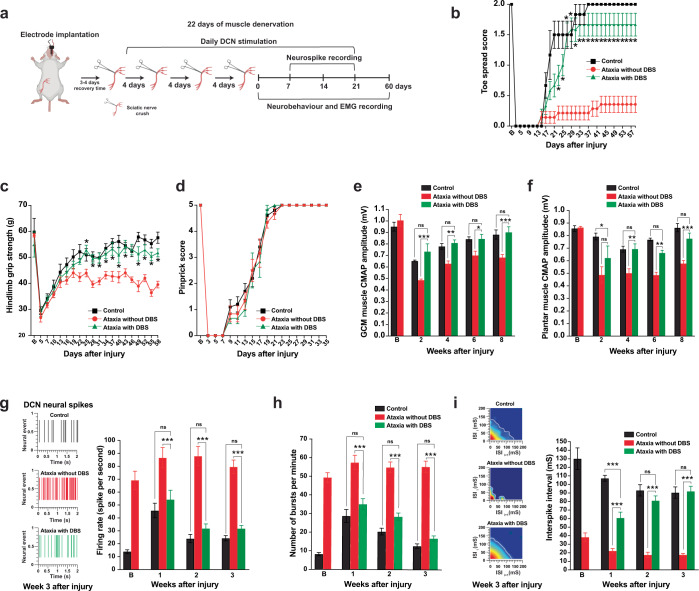
Deep cerebellar nuclei deep brain stimulation (DCN-DBS) restores cerebellar circuitry and recovers motor function recovery after multiple sciatic nerve crush (SNC) injury. **a** Schematic diagram illustrating the experimental paradigm for the animal model of 22 days of muscle denervation and DCN-DBS treatment. Daily 1-h DCN-DBS was started after the first SNC for 33 consecutive days. **b** and **c** Daily DCN-DBS reversed the motor deficits in ataxia mice, while ataxia mice without DBS were not able to fully restore motor function after SNC. **d** Recovery of sensory function was not affected in ataxia mice with or without DCN-DBS, when compared with control mice. **e** and **f** Ataxia mice with DCN-DBS recovered compound muscle action potential (CMAP) amplitudes from proximal and distal muscles gradually which were comparable to the control levels. **g** and **h** DCN neural firing rate and number of bursts in ataxia mice with DCN-DBS were indistinguishable from controls throughout the assessment period. Representative DCN neural firing patterns were shown in the left panel of **g**. **i** Average interspike intervals (ISI) of ataxia mice were increased gradually and comparable to control from week 2 post-SNC onwards. Representative ISI distribution heat maps were shown in the left panel. *n* = 8–10 mice per group for neurobehavioral experiments in **b**–**d**; *n* = 6–8 mice per group for EMG recording (**e** and **f**) and DCN neurospike firing analysis (**g**–**i**). Values represent the mean ± SEM; two-way ANOVA followed by post hoc Bonferroni test; *P < 0.05; **P < 0.01; ***P < 0.001. ns; non-significant. Detailed statistics analyses comparing CMAP amplitude data points across time within groups are available in Supplementary Table 2.

We then performed DBS targeting interposed nucleus of DCN to restore neural firing pattern in ataxia mice followed by SNC and behavioral tests. Mice received 1 h of daily DCN-DBS at 130 Hz/100 µA/80 µs^51,52^ using multielectrode arrays for 21 consecutive days following a single SNC (Supplementary Fig. 8a). We found persistent aberrant neural firing in ataxia mice during the course of recovery. By contrast, DCN-DBS restored the cerebello-cortical network by reducing the aberrant neural firing (Supplementary Fig. 8b) and the number of bursts (Supplementary Fig. 8c) and increasing the interspike interval (Supplementary Fig. 8d) as early as one week after DCN-DBS. Ataxia mice with DCN-DBS and control mice reached full toe spreading reflex, and restored grip strength and EMG activities in a comparable manner without any delays. However, implanted ataxia mice without DCN stimulation exhibited significant delayed full toe spreading reflex, and reduction of grip strength and EMG activities at 1-month post-SNC, while ataxia mice with DCN-DBS and control mice were fully recovered (Supplementary Fig. 8e–h). The sensory function recovery was unaffected in all three treatment groups (Supplementary Fig. 8i). Next, we examined whether DCN-DBS could recover motor function in ataxia mice within the critical period^1,7^, in which ataxia mice shorten the critical period from 37 to 22 days and failed to recover motor function after 22 days of muscle denervation (Supplementary Fig. 4). Mice received 1 h daily electrical stimulation for 33 consecutive days after the first crush (Fig. 4a). Implanted ataxia mice without DCN-DBS showed incomplete motor function recovery. In stark contrast, motor and sensory function recovery (Fig. 4b–d), EMG activities (Fig. 4e, f), and DCN neural firing properties (Fig. 4g–i) in ataxia mice with DCN-DBS were indistinguishable from control mice throughout the assessment period after multiple SNCs.

**Fig. 5:**
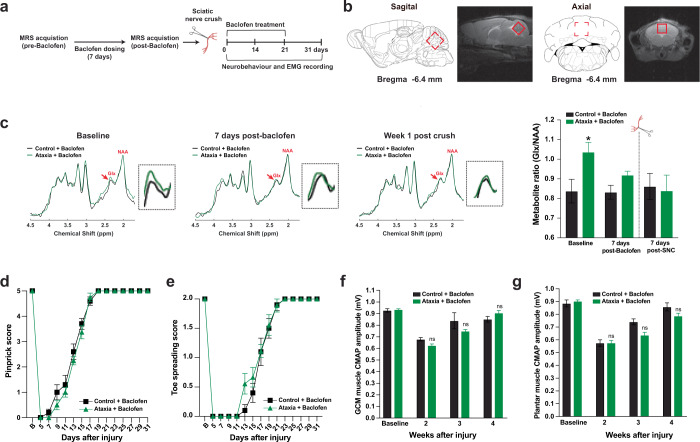
Baclofen reverses neurotransmitter imbalance as detected by magnetic resonance spectroscopy (MRS) and recovers spontaneous motor function after injury. **a** Schematic diagram illustrating the experimental paradigm for baclofen treatment and MRS acquisition. **b** Sagittal and axial T_2_ weighted images with the voxel of interest placed within the cerebellum (red squares). MRS data were analyzed by a customized MATLAB program. Schematic illustrations of sagittal and coronal sections were adopted from the Allen Mouse Brain Atlas (Reference Atlas Version 1, 2008). **c** Representative MRS spectra of control and ataxia mice were shown at different timepoints. MRS spectra were aligned by setting N-acetylaspartate (NAA) peak at 2.2 parts per million (ppm) and glutamate-glutamine (Glx) at 2.34 ppm as shown by red arrows. Glx level in ataxia mice was significantly higher than in control mice and restored to baseline levels of control mice after one week of baclofen treatment. **d**–**g** Ataxia mice treated with baclofen showed a similar level of restoration of sensory and motor function, and EMG activities, compared to control mice after SNC. *n* = 5 per group for MRS experiments (**b** and **c**). *n* = 9–10 mice per group for neurobehavioral experiments and EMG recording (**d**–**g**). Values represent the mean ± SEM; two-way ANOVA followed by post hoc Bonferroni test except in L, unpaired Student’s *t*-tests were used; *P < 0.05; **P < 0.01, ***P < 0.001. ns non-significant. Detailed statistics analyses comparing compound muscle action potential (CMAP) amplitude data points across time within groups are available in Supplementary Table 2.

Next, we examined the level of excitatory neurotransmitters such as glutamate-glutamine (Glx) in the cerebellum using non-invasive MRS during the course of baclofen treatment and recovery from a single SNC (Fig. 5a, b)^53,54^. Glx level in ataxia mice was significantly higher than control mice and restored to baseline levels after one week of baclofen treatment. Both prolonged baclofen treatment and SNC did not affect the Glx level in control mice (Fig. 5c). To ensure the complete restoration of Glx levels in baclofen-treated ataxia mice, MRS quantification of Glx level in the cerebellum was performed before we subjected the mice to SNC and subsequent functional recovery tests. Baclofen-treated ataxia mice, with Glx level, returned to the baseline levels of control mice, and showed a similar level of restoration of sensory and motor function (Fig. 5d, e) and EMG activities (Fig. 5f, g), compared to control mice after SNC. A separate group of mice was used for electrophysiology studies and toe spreading reflex tests were performed to examine motor recovery after SNC. Similar to DCN-DBS treatment, baclofen restored neural firing pattern, and the neural firing rate remained relatively steady in both treatment groups throughout the 21-days baclofen treatment and animal behavioral assessment period (Supplementary Fig. 9a, b). The number of bursts (Supplementary Fig. 9c) and interspike interval in ataxia mice (Supplementary Fig. 9d) returned to the level of control mice starting on days 7 post-baclofen treatment and sustained to week 3 in which all the mice were fully recovered. Collectively, these data suggest restoring cerebellar circuitry integrity and neurotransmitter imbalance and recovering spontaneous motor function after the PNIs.

### The mechanistic roles of glutamate receptor in spontaneous motor recovery after PNI

To gain further mechanistic insight into the roles of glutamine in the recovery of spontaneous motor function after PNIs, we examined the transcriptomic changes of a number of key glutamate receptor family members including the AMPA (Gria1, Gria2, Gria3, and Gria4) and N-methyl-d-aspartate (NMDA) (Grin1, Grin2a, Grin2b, Grin2c, Grin3a, and Grin3b) receptors in DCN. Compared to the uninjured mice, we found that the mRNA expression levels of *Gria3*, *Gria4*, *Grin2b*, and *Grin2c* were markedly increased in DCN at day 7 post-SNC in non-ataxia control and ataxia mice (Supplementary Fig. 10a, b). Interestingly, mRNA expression of *Gria1* was upregulated in control mice but remained unchanged in ataxia mice after SNC, suggesting a potential role in spontaneous motor recovery of ataxia mice after PNIs.

**Fig. 6:**
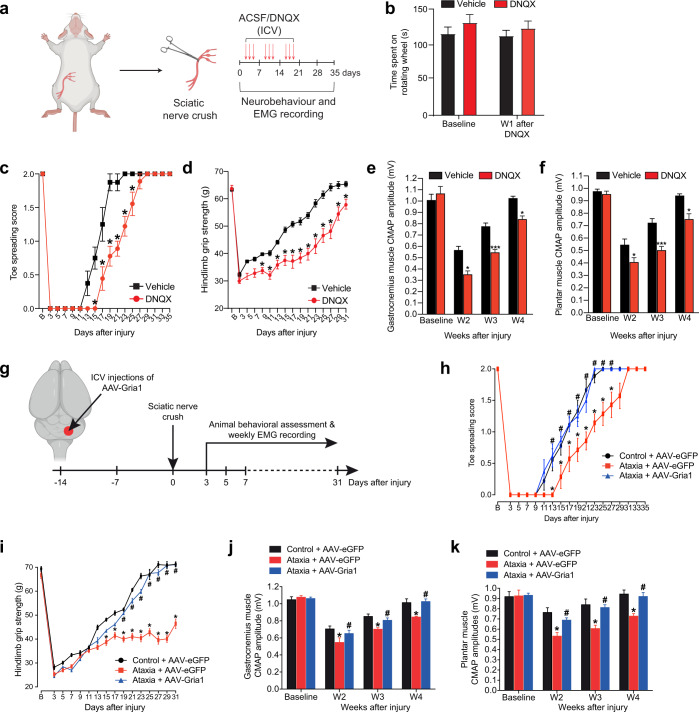
Gria1 is required for spontaneous motor recovery in ataxia mice after PNI. **a** Schematic diagram illustrating the experimental paradigm for DNQX treatment and neurobehavior assessment after a single sciatic nerve crush (SNC). Artificial cerebrospinal fluid (vehicle control) or DNQX was intracerebroventricular injected 3 times a week through a cannula implanted into the DCN. **b** The retention time on rotarod in DNQX-treated mice was comparable with vehicle-treated controls, suggesting that motor coordination was not affected by DNQX treatment. **c** Pharmaceutical blockade of AMPA receptors using DNQX severely impaired motor recovery as assessed by toe spreading test. The complete restoration of toe spreading reflex was markedly delayed by 6 days in DNQX-treated mice, compared with those treated with vehicle controls. **d** More drastically, the hindlimb grip strength of DNQX-treated mice was significantly lowered from days 9 to 31 post-injury, while vehicle-treated mice showed gradual and complete restoration of hindlimb grip strength. **e** and **f** The compound muscle action potential (CMAP) amplitudes of proximal gastrocnemius (**e**) and distal plantar muscle (**f**) were reduced in DNQX-treated mice compared with vehicle-treated controls. **g** Two weeks before SNC, ataxia mice received an intracerebroventricular injection of AAV-Gria1 or AAV-eGFP (control) into the cerebellum. Animal behavioral assessments were performed on every alternate day starting from day 3 after crush. **h** and **i** Ataxia mice treated with AAV-Gria1 showed complete motor recovery similar to the control mice injected with AAV-eGFP. **j** and **k** The CMAP amplitudes of proximal gastrocnemius (**j**) and distal plantar muscle (**k**) from ataxia mice treated with AAV-Gria1 restored to baseline level of controls. Values represented the mean ± SEM (*n* = 9–10 per group in **b**–**f**); (*n* = 7 for ataxia + AAV-eGFP group; *n* = 9 for control + AAV-eGFP and ataxia + AAV-Gria1 groups in **h**–**k**); *P < 0.05; ***P < 0.001, compared with vehicle-treated controls in **c**–**f**, or control mice with AAV-eGFP in **h**–**k**; # *P* < 0.05, compared with ataxia mice with AAV-eGFP in **h**–**k**, two-way ANOVA with repeated measures followed by post hoc Bonferroni test. Detailed statistics analyses comparing CMAP amplitude data points across time within groups are available in Supplementary Table 2.

To characterize the functional role of *Gria1* in PNIs, Gria1 was blocked pharmacologically in wild-type C57BL/6 mice using DNQX^55^, a selective antagonist of the ionotropic glutamate receptors, AMPA receptors are the most abundant ionotropic glutamate receptors in the brain. DNQX was administered through a cannula implanted into the DCN immediately after the sciatic nerve crush for 3 weeks (3 injections a week on alternate days) and functional recovery tests were performed on alternate days (Fig. 6a). We first assessed the baseline motor coordination of DNQX-treated mice by accelerated rotarod test and DNQX treatment did not induce ataxia-like motor coordination defects as we observed in naïve ataxia mice (Fig. 6b). The sensory and motor baseline values were comparable between vehicle-treated and DNQX-treated mice. However, the full toe spreading reflex of DNQX-treated mice was delayed until 29 days post-injury, while vehicle-treated mice were fully recovered on day 23 (Fig. 6c). Motor function was further quantified by measuring the grip strength of the hindlimbs. The hindlimb grip strength of DNQX-treated mice was significantly lower than the vehicle-treatment mice from 9 to 31 days post-injury and regained partial grip strength by 31 days post-injury (Fig. 6d). EMG recordings in the proximal (gastrocnemius) and distal (plantar) muscles showed that vehicle-treated mice recovered much faster than the DNQX-treated mice. Delayed and incomplete recovery in muscle activities were observed in the DNQX-treated mice, reaching up to 83% of the recovery in the most distal plantar muscle compared with uninjured baseline CMAP values (Fig. 6e, f). As expected, the recovery of sensory function was unaffected in DNQX-treated mice as assessed by pinprick assay (Supplementary Fig. 10c).

Finally, we asked whether increasing the expression level of *Gria1* in the DCN of ataxia mice could rescue motor deficits in the ataxia mice after SNC. Two weeks before the SNC, adeno-associated virus serotype 2 (AAV2) overexpressing mouse *Gria1* (AAV-Gria1) (Supplementary Fig. 10d) was intracerebroventricularly injected into the DCN of ataxia mice (Fig. 6g). Compared with AAV-eGFP-treated ataxia mice, we observed a nearly 3-fold increase in mRNA expression of *Gria1* in the DCN of AAV-Gria1-treated ataxia mice (Supplementary Fig. 10e). Strikingly, overexpression of *Gria1* completely restored motor deficits in ataxia mice and comparable to that in the AAV-eGFP-control mice after a single SNC, while the AAV-eGFP-treated ataxia mice were unable to return to baseline motor function (Fig. 6h–k). No difference in sensory function recovery was detected in these three mouse groups after SNC (Supplementary Fig. 10f).

### GEO database analysis reveals a downregulation of *GRIA1* in patients with ataxic manifestations

**Fig. 7:**
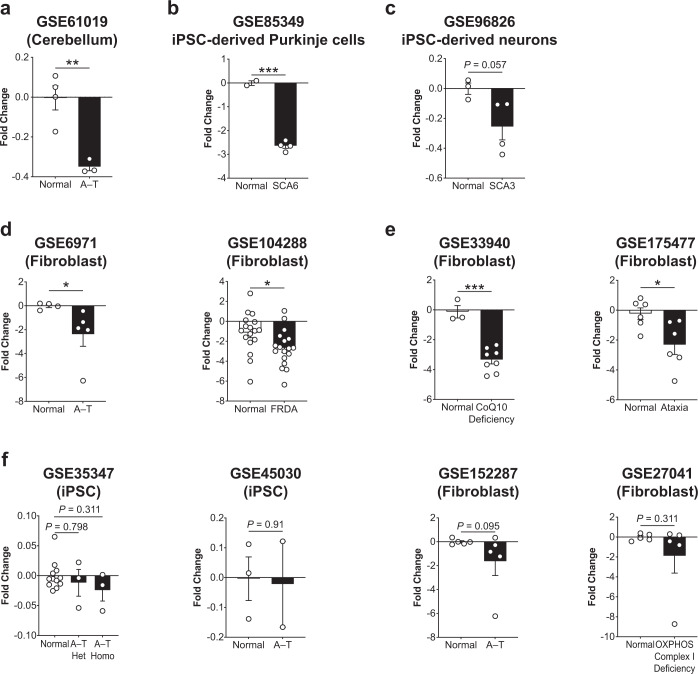
GRIA1 expression is downregulated in patients with ataxic manifestations. **a** The mRNA expression of human GRIA1 was significantly reduced in the cerebellum of human patients with ataxia-telangiectasia (A-T). **b** and **c** Similarly, GRIA1 was downregulated in the patient iPSC-derived Purkinje cells (spinocerebellar ataxia 6) and neurons with (spinocerebellar ataxia 3). **d** and **e** GRIA1 expression were markedly reduced in the fibroblast cells derived from patients with A-T and Friedreich’s ataxia, and patients with ataxia symptoms. **f** There was a trend for GRIA1 to decrease in A-T patient-derived iPSC and fibroblasts, and fibroblast derived from patients with oxidative phosphorylation disease who exhibited ataxic manifestations. Values represent the mean ± SEM. *P < 0.05, **P < 0.01, ***P < 0.0001; unpaired Student’s *t*-test for comparison between two groups, or one-way ANOVA followed by post hoc Bonferroni test for comparison between more than two groups.

Next, we aimed to evaluate the relevance of the mouse model of ataxia to understanding the motor function deficits associated with ataxia patients after PNIs. We systematically examined the mRNA expression profiles of GRIA1 in 11 publicly available ataxia patient gene expression datasets deposited in GEO, including human microarray and RNA-seq transcriptomic expression data of cerebellum, patient iPSC-derived Purkinje cell and neuron, and patient-derived fibroblasts. We found that there was a substantial reduction of mRNA expression of human GRIA1 (GSE61019: log2 fold-change of −0.35 ± 0.02, *P* < 0.01; unpaired Student’s *t*-test) in the cerebellum of patients with ataxia-telangiectasia (A-T), compared with the age-matched healthy individuals (Fig. 7a)^56^. Similarly, spinocerebellar ataxia type 6 (SCA6) patient iPSC-derived Purkinje cells also showed significant downregulation of GRIA1 (GSE85349: log2 fold-change of −2.65 ± 0.10, *P* < 0.0001; unpaired Student’s *t*-test), when compared with Purkinje cell derived from healthy individuals (Fig. 7b)^57^. There was a marginal non-significant trend for the downregulation of GRIA1 in SCA3 patient iPSC-derived neurons (GSE96826: log2 fold-change of −0.26 ± 0.09, *P* = 0.057; unpaired Student’s *t*-test), when compared with healthy human iPSC-derived neurons (Fig. 7c)^58^. Significant downregulation of GRIA1 was detected in A-T (GSE6971: log2 fold-change of −2.39 ± 1.01, *P* < 0.05; unpaired Student’s *t*-test)^59^ and Friedreich’s ataxia (GSE104288; log2 fold-change of −2.68 ± 0.43; *P* < 0.05; unpaired Student’s *t*-test)^60^ patient-derived fibroblasts compare to healthy controls (Fig. 7d). We also found that the mRNA expression of GRIA1 in the fibroblasts derived from patients with coenzyme Q10 deficiency or mitochondrial DNA m.3243 A > G mutation, who developed typical clinical cerebellar ataxia symptoms, was significantly lower than that in healthy donor-derived fibroblasts (GSE33940: log2 fold-change of −3.35 ± 0.27, *P* < 0.0001; unpaired Student’s *t*-test; GSE175477: log2 fold-change of −2.32 ± 0.65, *P* < 0.05; unpaired Student’s *t*-test) (Fig. 7e)^61^. We noted a slight trend for GRIA1 to decrease in A-T patient-derived iPSCs (GSE35347: log2 fold-change of −0.024 ± 0.018, *P* = 0.311; one-way ANOVA followed by post hoc Bonferroni test; and GSE45030: log2 fold-change of −0.022 ± 0.1440, *P* = 0.91; unpaired Student’s *t*-test)^62,63^, A-T patient-derived fibroblast (GSE152287: log2 fold-change of −1.65 ± 1.18, *P* = 0.095; unpaired Student’s *t*-test)^64^ as well as fibroblast derived from patient with oxidative phosphorylation disease, who exhibited a wide variety of clinical manifestations including ataxia (GSE27041: log2 fold-change of −1.90 ± 1.72, *P* = 0.311; unpaired Student’s *t*-test) (Fig. 7f)^65^. Together, these data indicate that GRIA1 plays an important role in spontaneous motor function recovery after PNIs, especially in patients with clinical manifestations that include ataxia.

### Ataxia patients fail to recover from carpal tunnel decompression surgery or tibial neuropathy

**Fig. 8:**
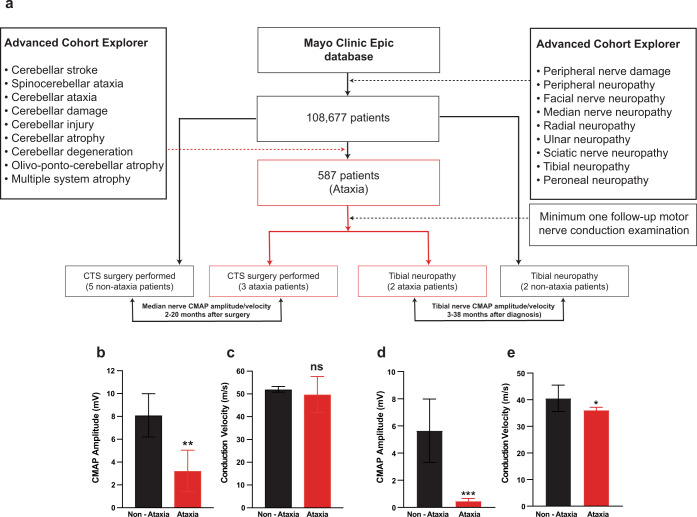
Ataxia patients fail to recover motor nerve conduction after carpal tunnel decompression surgery or tibial neuropathy. **a** Flow diagram illustrating patient study population selection. Utilizing the Mayo Clinic Epic electronic database, 12 patients were identified for motor nerve conduction data analysis. **b** and **c** Median nerve compound muscle action potential (CMAP) motor amplitude and conduction velocity after 2–20 months of carpal tunnel syndrome (CTS) surgery in ataxia (*n* = 3) and non-ataxia (*n* = 5) patients. Ataxia patients recovered the median nerve conduction to a lesser extent even after carpal tunnel decompression surgery. **d** and **e** Tibial nerve CMAP motor amplitude and conduction velocity after 3–38 months of tibial neuropathy in ataxia (*n* = 2) and non-ataxia (*n* = 2) patients. Non-ataxia patients tend to spontaneously recover full motor function during a comparable period of time for both groups of patients. Values represent the mean ± SD (standard deviation); *P < 0.05; **P < 0.01; ***P < 0.001, unpaired Student’s *t*-test.

We next sought to investigate the spontaneous motor function recovery in ataxia patients after PNIs by conducting a large-scale analysis of 9,655,320 patients from the Mayo Clinic Epic electronic database (non-public). The initial search identified 108,677 patients with PNIs and 587 of them have been diagnosed with cerebellar ataxia. Patients with median or tibial neuropathy were selected since they both are the most commonly encountered PNIs in humans. Patients with median neuropathy at the wrist (also known as carpal tunnel syndrome, CTS) are curable by surgical intervention (carpal tunnel release procedure), and usually no treatment is needed for most tibial neuropathy cases. Only the patients with precise date of peripheral nerve lesion diagnosis and those with at least one follow-up visit with a proper nerve conduction study done after the surgical intervention (for CTS patients) or being diagnosed with tibial neuropathy, were selected for analysis (Fig. 8a). A total of 12 patients met the inclusion criteria, 7 controlled non-ataxia patients with no history of cerebellar damage (3 females and 4 males, 28–77 years old, mean age 58 years), and 5 patients with cerebellar ataxia (1 female and 4 males, 49–76 years old, mean age 64 years).

For patients with CTS, 10 nerve conduction studies (NCSs) were conducted on non-ataxia patients and 6 NCSs were done on ataxia patients after surgery. The median nerve CMAP amplitude of non-ataxia patients returned back to normal values of 8.1 ± 1.89 mV (mean ± SD for all clinical data) in 2–20 months after CTS surgery (normal median CMAP amplitude is >4 mV^66,67^); however, ataxia patients recovered to a lesser extent (3.2 ± 1.80 mV; 39.5% of non-ataxia patients) after carpal tunnel decompression surgery (Fig. 8b). The motor nerve conduction velocity (ataxia: 52.0 ± 1.26 m/s; non-ataxia: 49.8 ± 7.88 m/s) was comparable between two groups (Fig. 8c). Our clinical data indicated that ataxia patients were unable to fully recover motor function after CTS surgical treatment when compared with non-ataxia patients over the same timeframe of recovery.

Tibial CMAP amplitudes were measured in patients from 3 to 38 months after being diagnosed with tibial neuropathy, in which injured neurons are required to regenerate their axons over a relatively long distance before they could reinnervate the target muscles. The mean tibial CMAP amplitude of ataxia patients was 0.45 ± 0.19 mV (nNCS = 8), which was much lower than the non-ataxia patients (5.65 ± 2.33 mV, nNCS = 2) (Fig. 8d). In one of the non-ataxia patients, two additional NCSs were conducted at the 61-month (6.6 mV) and 86-month (6.1 mV) visits after being diagnosed with tibial neuropathy. In ataxia patients, we observed a slowing of tibial motor nerve conduction velocity (36 ± 1.15 m/s), when compared with non-ataxia patients (40.5 ± 4.95 m/s) (Fig. 8e). These clinical data suggest that ataxia patients with tibial nerve lesions fails to recover motor function, while non-ataxia patients tend to spontaneously recover full motor function during a comparable period of time for both groups of patients. In line with the single and multiple SNC results in ataxia mice, the distance of axonal regrowth might be positively correlated with the extent of irreversible motor deficit in ataxia patients.

## Discussion

Our results establish a causal relationship between cerebellar circuitry integrity and motor recovery after PNIs. The study of distinct mouse models and ataxia patients with PNIs demonstrates the increased risk of motor deficits associated with cerebellar dysfunction after PNIs. The MRS findings of elevated Glx levels in the cerebellum of ataxia mice suggests the possibility of their connection to the disruption of glutamatergic system. We further demonstrated that by restoring cerebellar circuitry using cerebellar DBS or gamma-aminobutyric acid (GABA_B_) receptor agonist baclofen, we were able to reverse motor deficits in ataxia mice after PNIs. mRNA expression of key glutamine receptors were upregulated in both control and ataxia mice after PNI except *Gria1*. AAV-mediated cerebellar gene delivery of *Gria1* was able to rescue the motor deficits of ataxia mice after PNI. We systematically identified downregulated human GRIA1 expression in patients with ataxic manifestations across several human GEO datasets. These findings, in line with our clinical data, reflect what happens in ataxia patients after PNIs. We demonstrate a concept that ataxia patients, which has a 93% chance of having at least one fall in a year^68^, could be more prone to have irreversible motor deficits whereas the majority of non-ataxia patients are able to recover spontaneously from PNIs such as CTS and tibial neuropathy.

We first set out to explore the possibility that aberrant hyperexcitability of cerebellar DCN is the primary cause of poor motor recovery after PNIs in ataxia mice and found that disruption of cerebellar circuitry severely affects motor recovery. DCN function as a relay center for inhibitory inputs from Purkinje cells in the cerebellar cortex and excitatory inputs from mossy and climbing fibers, and project to various premotor areas to fine-tune motor activity. Abnormal high frequency burst neural firing pattern in DCN has been linked to ataxia, dystonic Parkinsonism, and tremor^47,69,70^. Recent studies demonstrate the interposed nucleus as a DBS target for restoring aberrant neural firing in the DCN and potential treatment of tremors^47^ and ataxia^50^. Notably, a long-lasting beneficial effect is observed in the ataxia mice after daily DBS (20–40 min DBS a day for 4 consecutive days)^50^. In Parkinson’s disease patients, it has been demonstrated that switching off a DBS would not stop the beneficial effect abruptly^71,72^. Functional MRI studies show that there is a remarkable long-term effect of DBS on structural and functional connectivity (brain rewiring or neuroplasticity)^73^. Our electrophysiology data showed that DBS of DCN normalized aberrant neural firing leading to complete motor recovery in ataxia mice after a single and multiple SNC, whereas we performed DCN-DBS for 21 consecutive days which is the time period for active axon regeneration and function recovery^1,7,37,74^. Therefore, we show that an abnormal pattern of activity that is transmitted from Purkinje cells downstream to the DCN in ataxia mice can be restored for better motor recovery from PNI.

GABA is the primary inhibitory neurotransmitter in the CNS and abnormalities in GABAergic system have been widely reported in ataxia^75–78^. Purkinje cells are GABAergic neurons and the GABAergic axons of the Purkinje cells that form synapses on DCN are greatly reduced in our ataxia mice as a result of dendritic thinning^23^. The removal of inhibition of excitatory synapses due to the reduction of GABAergic inhibition has been suggested to be the most important cause of acute neuroplasticity changes in the cerebellum^79^. In addition, peripheral deafferentation often results in a reduction in the number of neurons containing GABA as well as the glutamic acid decarboxylase (GABA synthesizing enzyme) in the cortical area^80,81^. In humans and primates, finger deafferentation by anesthetic block induces not only rapid functional changes but also the partial expansion of the cortical representations of intact fingers into the cortical map of denervated finger within minutes^82,83^. Consistent with these studies, we found that by restoration of cerebellar circuitry through administration of baclofen, a clinically available GABA_B_ receptor agonist^84^, restored normal neural firing pattern of DCN and recovered motor function in ataxia mice after SNC. Baclofen treatment also restored Glx level in the DCN of ataxia mice as determined by using noninvasive MRS. In fact, there is an increasing interest in the roles of brain metabolites such as Glx in spinal cord injury and neuropathic pain since glutamate and glutamine are relatively abundant amino acids in the brain that are critical for neuronal function^85^. It has been shown that an elevated level of Glx in the anterior cingulate cortex is associated with SCI-related neuropathic pain in patients^86^. In rats, Glx decreases dramatically in the cortex and gradually returns to normal levels after a low thoracic spinal cord injury^87^.

In this study, we report for the first time that there is a potential mechanistic link between Gria1 and spontaneous motor recovery in patients with ataxia due to the glutamatergic dysfunction. GRIA1 encodes the GluA1 subunit of the AMPA subtype of glutamine receptor, which is an essential component of synaptic plasticity, especially for learning and memory functions in AD. Disruption of AMPA receptor trafficking by Abeta oligomers in the hippocampus is believed to be a major contributor to synaptic dysfunction in AD, leading to cognitive deficits^88^. Purkinje cells express all four AMPA receptor subunits GluA1-4^89,90^, and intense GluR1 immunoreactivity and mRNA expression are detected in the Purkinje cells^91,92^. We therefore speculate that synaptic dysfunction associated with downregulation of cerebellar Gria1 in ataxia mice and patients with ataxic manifestations, leading to motor deficits after PNI. PNI induces not only substantial changes in neural plasticity in the CNS^13,93^ but also transcriptomic changes in a number of key genes involved in the regulation of synaptic plasticity^13^. There is growing evidence that *Gria1* expression and the balance of glutamatergic throughput via AMPA receptors are crucial for neural plasticity^94,95^. In rats, PNI up-regulated gene expression of ion channels, AMPA receptors (*Gria1, Gria3*, and *Gria4*), GABA receptors, and signaling transduction molecules in the dorsal spinal cord^96–98^. Co-deletion of two AMPA receptor subunits *Gria1* and *Gria4* in cerebellar Bergmann glial cells, leads to impairments in fine motor coordination^99^. To this end, we conclude that an intact cerebellar circuitry and a proper AMPA receptor-mediated glutamatergic neurotransmission are crucial pre-requisite to the success of motor function recovery after PNIs. In line with our hypothesis, the interruption of the hippocampal circuit (non-motor performance brain area) does not affect spontaneous motor recovery indicating that synaptic integrity of the glutaminergic system in the cerebellum is necessary for complete motor recovery after PNI. Future research examining the temporal correlations between glutamatergic neurotransmission and motor recovery would be useful to better understand the links between brain circuitry and PNS regeneration.

The PNS and CNS are functionally integrated that PNI induces profound and permanent CNS modification and reorganization^9–12^. Remodeling of spinal and brain circuits after PNIs usually do not allow for a meaningful functional recovery, especially after sever PNIs^13^. The conceptualization of improving CNS plasticity for better functional recovery in the PNS has been emerged for decades^14^. Promoting spinal plasticity by chondroitinase improve recovery of forelimb function after PNI^15^. Our work on the single and multiple SNC, that adult mice usually take 4–8 weeks to regain full motor function^1,7,37,74^, suggests that disruption of cerebellar circuitry leads to permanent motor deficits. This raise the intriguing possibility that the major cause of motor deficits in patients with cerebellar disorders might not be the diseases themselves but perhaps the unrecognized PNI sustained by the patients. Our clinical data support these notions are correlational, the patients with ataxia did not reach full motor recovery after carpal tunnel decompression surgery or recover spontaneously after tibial nerve neuropathy as observed in the non-ataxia patients (Fig. 8).

Although peripheral nerves can regenerate after injury, CNS reorganization (totally or partially) occurs depending on the severity of the injury and the distance of axon regeneration to accurately reinnervate their original targets. Our multiple SNC model, a form of severe PNI, extends the traveling distance as well as the time for regenerating axons to reach the original targets^1,7^. Ataxia mice were unable to perform voluntary motor tasks such as toe spreading reflex and displayed a reduction of grip strength by more than 50% (Fig. 1j, k). In patients with ataxia, the tibial nerve CMAP amplitude was 13 times lower than the non-ataxia patients (Fig. 8d). These data suggest that PNI-induced maladaptive plasticity becomes more pronounced with abnormal cerebellar cortex in ataxia and this possibility must be examined in detail in future studies.

In summary, we believe that it is imperative to raise awareness among healthcare providers which concerns not only the link between ataxia and irreversible motor deficits but also has additional implications in the context of movement disorders associated with cerebellar dysfunctions or physical damage to the cerebellum that might cause disruption to the glutamatergic system and descending motor circuits results in poor clinical outcomes following PNIs. One potential therapeutic strategy is to stimulate AMPA receptors, given that the clinical application of AMPA receptor potentiators has been reported for treating psychiatric diseases. A recent clinical trial demonstrated that AMPA receptor potentiator (PF-04958242) attenuated the schizophrenia-relevant ketamine-induced cognitive impairment in verbal learning and working memory^100^, suggesting the use of AMPA receptor potentiator for the treatment of cognitive impairments associated with schizophrenia. Additionally, PF-04958242 has been shown to improve auditory function significantly in age-related sensorineural hearing loss^101^. Another AMPA receptor potentiator, LY451395, which has been in Phase II development with possible use in Alzheimer’s disease whereas abnormalities of glutamatergic homeostasis also occur. A significant improvement in the neuropsychiatric symptoms was evident in patients with Alzheimer’s disease^102^. In mice, enhancement of AMPA receptor signaling by AMPA receptor agonists (CX 1837 and CX 1739) induced brain-derived neurotrophic factor secretion in periinfarct cortex and promoted motor function recovery after ischemic stroke. In contrast, pharmacological blocking of AMPA receptor signaling by AMPA receptor antagonist (CFM2) worsened motor task performance that normally recovers after stroke^103^. A novel AMPA receptor potentiator, TAK-137, not only enhanced cognitive function in naïve rats but also improved psychostimulant-induced hyperlocomotion^104^. These studies collectively suggest that the restoration of AMPA receptor function is critical to the maintenance of glutamatergic homeostasis that might show similarly favorable therapeutic benefits to improve motor function in patients with cerebellar dysfunctions and damages, and therefore warrant further investigation in a clinical setting.

## Methods

### Animals

All animal experimental protocols were approved by the Animal Research Ethics Sub-Committee at the City University of Hong Kong (ref. A-0017) and in compliance with the American Veterinary Medical Association (AVMA) Guidelines. Mice had free access to food and water and were maintained on a 12:12-h light–dark cycle. We made our best effort to reduce the number of animals used in current study. To generate Lhx1/5 double knockout mice, we crossed *Pcp2*-Cre mice (*Pcp2*-*Cre*^+/−^) with *Lhx1* fl and *Lhx5* fl (*Lhx1*^*fl*/fl^/*Lhx5*^*fl*/fl^) mice to obtain *Pcp2*-*Cre*^+/−^/*Lhx1*^fl/fl^/*Lhx5*^fl/fl^ (ataxia mice) and *Pcp2*-*Cre*^*−*^^/−^*/Lhx1*^fl/fl^/*Lhx5*^fl/fl^ (littermate mice) served as control mice^23^. Adult (8–10 weeks old) male double knockout mice and age-matched male littermates, and C57BL/6 wild-type adult mice were used for all experiments. Mouse lines were maintained on the C57BL6 background.

### Animal models of PNI and chronic muscle denervation

Adult male mice were anesthetized with 2.5% isoflurane, and the left sciatic nerve was crushed once with 5/45 smooth forceps (Fine Science Tools) for 15 s at the level of the external rotator muscles distal to the sciatic notch^1,2,6,7,37^. To delay muscle reinnervation for a specific period of time (16, 22, and 25 days), we crushed the sciatic nerves four times at 2-, 4-, and 5-day intervals for 16, 22, and 25 days of muscle denervation, respectively^1,7^. Animals were allowed to recover on heated pads before returning to their home cages. The surgeon who performed the surgery was blinded to the mouse genotypes and treatments.

### Sensory and motor function recovery tests

On day 3 after the last crush in single or multiple crush mice, we first performed a sensory pinprick assay followed by toe spreading reflex, grip strength measurement, and accelerated rotarod test every other day at least 30 min apart from each test^1,2,6,7,37^. The observers were blinded to the mouse genotypes, surgery, and treatment groups.

A sensory pinprick assay was performed to measure the successful regeneration of sensory axons into hindlimb skin, mice were placed on wire mesh cages and habituated for at least 30 min. An insect pin (FST) was gently applied from the most distal toe (score 5) to the heel (score 0) (divided into 5 areas), briskly withdrawal of ipsilateral hindlimb indicated a positive response. A score of 5 indicated a positive response from every tested area and complete recovery of sensory function. The area innervated by the saphenous nerve of the ipsilateral hindlimb was tested as a positive control if there was no positive response from these five areas.

To examine motor function recovery, we assessed spontaneous toe spreading reflex by covering the mice gently with a piece of cloth and picked up by the tail for clear observation. The toe spreading reflex was scored as: 0—no spreading; 1—intermediate spreading with all toes; and 2—full spreading was defined as a complete wide and sustained spreading of all toes for at least 2 seconds. Mice were scored when a full response was observed on the contralateral side to the injury. Mice were evaluated twice in each experimental session with at least a 45-min interval.

To determine the recovery of hindlimb grip strength, forelimbs were rested on a plastic bar and the hindlimbs were positioned to grip the metal T-bar, and gently pulled off. Values (in grams) at which the mice were released from the T-bar were recorded by a grip strength meter (BIO-GS3, Bioseb). Animals were trained for three alternative days to get accustomed to the grip strength machine before taking the baseline values. Five readings were taken from each mouse and average values were presented.

Motor coordination was assessed using the accelerated rotarod (Panlab 76-0772, Harvard apparatus) test. Mice were trained for 2 days at a constant (4 rpm) or accelerating speed (4–40 rpm in 5 min) for 2 min. Mice were placed on the rotating wheel and latency (retention time) for the mice to fall off from the rotating wheel was recorded in accelerated mode. A 15-min rest interval was provided between each set of 5 trials.

### EMG recording of gastrocnemius and interosseous (plantar) muscles

Mice were deeply anesthetized with ketamine (100 mg/kg) and xylazine (10 mg/kg) and placed on a heating pad to maintain body temperature. A monopolar needle (27-gauge) electrode for EMG recording was custom-made with stainless steel wire (A-M Systems, Sequim) and the impedance was tested before use (Gamry Instruments). Sciatic nerve stimulation (300 μA, 100 ms) was performed using a stimulator (Master 9; AMPI). The stimulating electrode was placed at the sciatic notch and the reference electrode was placed at the tail base. The EMG of the gastrocnemius muscle was recorded by inserting a recording electrode into the belly of gastrocnemius muscle and an Achilles tendon electrode as a reference. For EMG recording of interosseous muscle, the active and reference two-pin electrodes were inserted into the first and fourth interosseous muscles of the same hindlimb, respectively. EMG signals were sampled at 30 kHz (data acquisition set-up; Blackrock Microsystems), amplified, and bandpass-filtered with low and high frequency cutoffs of 10 and 250 Hz, respectively. The amplified EMG signal was then low-pass filtered (i.e., 2000 Hz) to remove aliasing in the 2000- to 3000-Hz range; any DC offset was also removed. CMAP amplitude was measured from the peak of the negative deflection to that of the positive deflection using Spike2. CMAP amplitude data are presented as mean ± SEM of 20 peaks/mice^6,7,37^.

### Sciatic nerve pinch test

A nerve pinch test was performed to quantify the rate of in vivo axon regeneration. Left sciatic nerves were crushed for 15 s^1,2^. Under light anesthesia (1% isoflurane), a series of pinches were delivered to the most distal part of the sciatic nerve and gradually moving proximally to the crushed site at 3 days post-injury. The distance (mm) from the injury site to the most distal point on the nerve that produces a reflex withdrawal when pinched was recorded. The distance represents the length of in vivo axon regeneration within 72 h from the crush site to the response site. The person who performed the nerve pinch test and the second observer who verified the reflex withdrawal, were both blinded to the mouse genotypes.

### Preconditioning primary DRG cultures and neurite outgrowth assay

Sciatic nerve transection was performed in adult control and ataxia mice by ligating the left sciatic nerve with 6-0 suture followed by complete transection distal to the ligature. Ipsilateral and contralateral L4 and L5 DRGs were harvested after 5 days (preconditioning) of nerve transection and primary dissociated neuronal culture was prepared^1,6^. Preconditioned DRGs were digested with collagenase/dispase II (Roche Diagnostics), trypsinized, and dissociated mechanically using Pasteur pipettes. A total of 1500 DRG neurons were plated onto 8-well chamber slides (Millipore) precoated with poly-d-lysine and laminin (Sigma-Aldrich). Neurobasal (NB) medium supplemented with B27, 200 mM l-glutamine, penicillin/streptomycin, 50 ng/mL nerve growth factor (Gibco), 2 ng/mL glial cell line-derived neurotrophic factor, and 10 μM cytosine arabinoside (Sigma-Aldrich) were used. The cultures were allowed to grow for 17 h and fixed with 4% paraformaldehyde (PFA), and immunostained with anti-β tubulin III antibody. Non-overlapping images were taken using a motorized fluorescence microscope (Nikon Eclipse 90i). Longest neurite length of individual neurons from each mouse genotype was measured by automated WIS-NeuroMath software and averaged from at least 180 neurons per genotype. Data were obtained from three separate experiments repeated in duplicates.

### DCN Lesion

Bipolar electrodes were prepared by perfluoroalkoxy-insulated stainless steel wires (AM systems, diameter 450 μm) and were positioned stereotaxically in the DCN of ketamine/xylazine anesthetized adult mice. Training and behavioral baselines were taken the week before the lesion. The electrodes were bilaterally inserted into the DCN at the following coordinates relative to bregma: anteroposterior (AP) = −6.4 mm, mediolateral (ML) = −1.3 mm, and dorsoventral (DV) = +2.5–3 mm from the skull surface^47^. Bilateral electrical lesions were produced by passing a current of 0.75 mA for 10 s. For the sham-operated mouse group, electrodes were placed in the DCN without passing any current. All animals were observed daily to monitor health and allowed 2 weeks to recover from the surgery. An accelerated rotarod test was performed to validate motor coordination deficits in the DCN lesion mice. The sensory and motor function were tested in the DCN-lesioned and sham-operated mice before performing the sciatic nerve injury, to confirm that both mouse groups have similar baseline values before the SNC.

### Hippocampal lesion

Adult mice were anesthetized with ketamine/xylazine and their heads were fixed using the stereotaxic apparatus. Animals were given multiple intracerebral infusions of NMDA) (Sigma-Aldrich)^43,44^. There were five infusions sites in each hemisphere centering on the hippocampus, which targeted CA1–CA3 area (see Table 1). Small openings were drilled in the exposed skull to perform bilateral infusions of either NMDA or sterile 0.9% phosphate buffer saline through a 1-μl Hamilton syringe connected to an automated syringe pump (Legato^®^ 130, KD Scientific). Lesion was produced by infusions of 0.1 μL of NMDA (10 mg/mL) over 1 min and the syringe was left in place for an additional 1 min before being slowly withdrawn. The mice were then allowed to recover for 3 weeks and were examined for spatial memory using the Morris water maze task and Y-Maze test. Baseline measurements of sensory and motor functions, EMG activities, and motor coordination (accelerated rotarod test), were taken from the hippocampus-lesioned mice with memory and learning deficits, and sham-operated mice for comparison.

**Table 1:** Stereotaxic coordinates employed for the injections of NMDA.

	Site 1	Site 2	Site 3	Site 4	Site 5
AP (mm)	−1.2	−1.7	−2.2	−2.7	−3.2
ML (mm)	±0.8	±1.0	±1.5	±2.5	±2.5
DV (mm)	−2.0	−2.0	−2.0	−2.4	−2.5

### Behavioral assessment of memory and learning

Three weeks after the hippocampal lesion, mice were subjected to Morris water maze task and Y-maze test. The same individual who handled the animals during the training period also performed all behavioral tests. The observer was blinded to the surgery and treatment groups.

Spatial memory and learning were assessed by using the Morris water maze. A round tank with a diameter of 150 and 60 cm deep was filled approximately half-way with water, and the water temperature was maintained at 25 °C. The maze was divided into four equal quadrants by two principal axes perpendicular to one another, and four visual cues were placed on the walls of the water maze room. Mice were trained for 5 days with 4 trials per day and a minimum of 15 min inter-trial interval for each mouse. During training, the starting position was alternated between trials but hidden platform was placed in the center of one of the quadrants. Mice were allowed to swim freely to a small hidden platform (10 cm in diameter) submersed 1.5 cm under the water surface. The platform was camouflaged by adding a non-toxic paint in the water and making it nearly invisible. This opaque color also provides an efficient background for the ANY-maze tracking system. If the mice did not find the hidden platform during a period of 90 s, it was gently guided to the platform, and allowed to remain on the platform for 15 s to get familiarize with their surroundings. A probe test was performed 24 h after the last training day to assess the learning memory by removing the platform and allowing the mice to swim freely for 90 s. The latency to reach the platform area was recorded and analyzed by the ANY-maze tracking system (Stoelting)^43^.

Spatial working memory was assessed by Y-maze test using a symmetrical Y-shaped maze with arms of equal length and angle. Each arm was 30 cm long, 10 cm wide, 20 cm height and interconnected with each other at a 120° angle. Y-maze arms were randomly divided into three arms—starting arm (SA), other arm (OA), and novel arm (NA). The test consisted of two trials of 5 min each with an inter-trial interval of 30 min. During the first trial, NA was blocked by a central partition and mice were placed at the end of SA, away from the center. The mice were allowed free access to the two arms (SA and OA) in the first trial and after that, it was returned to the home cage for the inter-trial interval. During the second trial, the central partition of NA was removed and the mice were allowed to explore all three arms. After each trial, the Y-maze was wiped with 75% ethanol to avoid the odor cues. During the experiment, the Y-maze was video recorded and the time spent in NA (%) were analyzed using the ANY-maze tracking software (Stoelting)^43^.

### Multielectrode arrays stimulation and recording of DCN in freely moving mice

Customized multielectrode arrays were fabricated with nichrome wire (AM systems, diameter 600 μm) and tested for impedance before use (Gamry instruments, USA). A 10-mm midline skin incision was made and bregma and lambda were located for precise stereotactic electrode placement. MEA electrodes were bilaterally inserted into the interposed nucleus of DCN at the following coordinates relative to bregma: AP = −6.4 mm, ML = −1.3 mm, and DV = +2.5–3 mm from the skull surface^47^. Screw and the MEA were affixed to the skull with dental cement (Megadental GmbH, Germany) to enhance the signal-to-noise ratio. The reference electrode was placed over the frontal bone. The exposed area of the skull was kept moist using artificial cerebrospinal fluid. The animals were observed daily and allowed to recover for 1 week after the surgery. This recovery period was based on the observation that no differences in motor behavior parameters were detected before and after electrode implantation at 5–7 days post-implantation.

Post-implantation electrophysiology and animal behavioral baselines were taken before the DCN-DBS. SNC was performed and electrode-implanted mice were divided into four groups: (1) Control mice with no DCN-DBS; (2) Control mice with DCN-DBS; (3) Ataxia mice with no DCN-DBS and (4) Ataxia mice with DCN-DBS. DCN stimulation (100 µA, 80 μs, 130 Hz) was performed for 1 h per day for 21 consecutive days after a single SNC, and for 33 consecutive days after the first SNC in the 22-day muscle denervation model. DCN stimulation (100 µA, 100 ms) at a frequency of 130 Hz was triggered using a stimulator (Master 9; AMPI).

Weekly MEA recordings of DCN were performed in both single and multiple SNC models after the sciatic nerve injury. Neural spike in DCN was recorded in freely-moving mice using the Apollo II acquisition system (Bio-Signal Technologies). Data were analyzed using Spike2 software, Blackrock offline spike sorting (BOSS) software (Blackrock Microsystem) for neurospike sorting. The sorted spike was further analyzed based on the principal component analysis (PCA) method to subtract error arising from electrode drifting and background signals. Our results showed good spike clustering and minimal data point drifting, which indicated low background noise levels. A customized MATLAB program was used to determine the frequency of neurospike, bursts, and interspike interval. We blinded the investigator who performed DCN neurospike data analysis by replacing the names of data files with a code name; this was done by a technician who was unaware of the mouse genotypes and treatments (with or without DCN-DBS).

### Baclofen treatment and in vivo MRS quantification of Glx level in cerebellum

Freshly prepared baclofen (10 mg/kg, Tocris Bioscience) was intraperitoneally injected into control and ataxia mice for 7 consecutive days before the SNC^105^. The Glx level in the cerebellum of the same mice was measured by MRS before baclofen treatment, 7 days after baclofen treatment, and 7 days after the SNC. Mice received a total of 21 consecutive days of baclofen treatment (Fig. 5a). Ataxia mice were used for SNC and subsequent functional recovery tests only if the Glx level returned to the baseline levels of control mice.

Magnetic resonance imaging was performed on a horizontal bore 3 T Bruker BioSpec system (Bruker). An 82 mm quadrature volume resonator was used as a transmitter, and a single surface coil was used for receiving signals. During magnetic resonance scanning, mouse anesthesia was induced and maintained using isoflurane at 2% and 1.5%, respectively. The sagittal and axial *T*_2_ weighted images were firstly acquired using rapid acquisition with refocused echoes (RARE) sequence, to localize a voxel of interest in cerebrum of each mouse for MRS data acquisition (see red square boxes in Fig. 5b). Care was taken to avoid cerebrospinal fluid spaces within the voxel of interests. Localized shimming up to second order was performed using voxel-based field mapping before each MRS acquisition. After field shimming, MRS data were acquired using the stimulated echo acquisition mode (STEAM) sequence with outer volume suppression. The acquisition parameters were: voxel size = 2 × 2 × 2 mm^3^, FID = 2048 points, averages = 256, TE = 3 ms, mixing time (TM) = 10 ms, TR = 2300 ms, scan time = 9 min 49 s. Water suppression was achieved using the variable pulse power and optimized relaxation (VAPOR) method^53,54^. MRS data processing and analysis were performed by MATLAB (MathWorks). MRS spectra were aligned by setting the N-acetylaspartate (NAA) peak at 2 parts per million (ppm). The peak areas for NAA at 2.02 ppm and Glx at 2.34 ppm were determined by Gaussian line shape fitting. The Glx to NAA ratio of each mouse brain was calculated for comparison.

### Electrical stimulation of DCN, motor cortex, and spinal cord with evoked EMG recording

We first determined the optimal stimulation current required to elicit EMG activities from DCN, motor cortex, and spinal cord^45–47^. A set of five stimuli was delivered for each stimulation current at 1 Hz and increased by increments of 25 µA ranging from 25 to 200 µA triggered by a stimulator (Master 9, AMPI, Israel) (Supplementary Fig. 11). The optimal stimulation current was determined as the stimuli which induced maximal EMG response before reaching a plateau. The input/output protocol was repeated in each individual mouse that was subjected to electrical stimulation to ensure reproducibility. Optimized stimulation parameters (100 µA current, 100 µs pulse width, 1 Hz frequency) was used for electrical stimulation of DCN, motor cortex, and spinal cord with evoked EMG recording. Electrical stimulation of DCN/motor cortex/spinal cord and EMG recording were performed simultaneously for 30 min. We performed electrical stimulation of DCN first and then followed by the motor cortex and spinal cord of the same mouse in a sequential manner. As described above, the MEA for DCN stimulation and monopolar needle electrode for EMG recording was implanted into the DCN and gastrocnemius muscle, respectively.

After electrical stimulation of DCN, the MEA electrode was removed and a 0.5 mm craniotomy was made over the representative hindlimb area of right motor cortex (1 mm posterior to bregma and 1.5 mm lateral) for the implantation of the MEA electrode. After repeating the input/output protocol, motor cortex was stimulated (100 µA, 100 µs, 1 Hz) with simultaneous recording of EMG for 30 min.

Lastly, the skull was closed by suturing the skin, and a laminectomy between T12 and L5 was carried out to exposed the lumbar spinal cord. MEA electrode was placed in the ventral horn of the spinal cord (0.5 mm lateral to the midline and 0.5 mm deep from the dorsal surface of the spinal cord) stereotaxically and covered with small cotton balls wetted with saline. A reference electrode was placed subcutaneously in the contralateral side of the recording electrode. After the input/output protocol for electrical stimulation of the spinal cord was validated, ventral horn of the spinal cord (near to L2–L5) was stimulated (100 µA, 100 µs, 1 Hz) with simultaneous recording of EMG for 30 min.

To calculate nerve conduction latency, the stimulation marker channel was superimposed with EMG response, and the time difference between electrical stimulation and EMG response was recorded. An average of 25–30 EMG responses for each mouse were used for CMAP amplitudes and latency calculation. The raw data of EMG recordings were examined by a second person who was blinded to the treatment groups.

### Implantation of cannula for intracerebroventricular administration of DNQX

Mice were anesthetized with ketamine/xylazine and placed on a stereotaxic surgery apparatus. A midline incision (10 mm) was made to locate the bregma as a reference. DCN (AP = −6.4 mm; ML = −1.3 mm; DV = +2.5 mm from the skull surface) position was located on the right cerebellum and a 0.5 mm craniotomy was performed. Dual cannula system for mice [RWD 62001; outer diameter (0.48 mm)×internal diameter (0.34 mm)] was secured in DCN using dental acrylic. One week after implantation, mice were randomly assigned into control and treatment groups.

Stock solution of DNQX (1.67 µg/µL, Tocris Bioscience) was prepared in artificial cerebrospinal fluid (ACSF; Tocris Bioscience)^106,107^. Aliquots were stored at −80 °C and diluted (10 times) to final concentrations in ACSF on the day of use. DNQX (1 µL) and ACSF (1 µL) were infused using a 26-gauge needle attached to a 10-µL Hamilton syringe at a slow rate of 0.2 µL/min (Pump11, Harvard Apparatus) and the syringe was left in place for an additional 5 min before being slowly withdrawn. Mice received three injections per week for 3 consecutive weeks after SNC.

### RNA extraction, reverse transcription and qPCR analysis

Total RNA was extracted from the cerebellum of injured mice 7 days after SNC using Trizol reagent (Takara), and the concentration of RNA was determined using Nanodrop 2000. Reverse transcription was performed using PrimeScript RT Reagent Kit with gDNA Eraser (Takara). qPCR analysis was performed in triplicate using TB Green Premix Ex Taq qPCR Kit (Takara) on a QuantStudio 12K Flex Real-Time PCR system. The Ct-value of each gene was recorded from each replicate and used to calculate the relative fold change using 2^−ΔΔCt^ formula, and *Gapdh* was used as the internal standard for the normalization^7,108^. The sequences of forward and reverse primers used in the study are listed in Supplementary Table 1.

### Intracerebroventricular injection of AAV

To overexpress ChR2 and mouse Gria1 in DCN, a midline incision (10 mm) was made to locate the bregma and lambda sutures for precise microinjection, and a 0.5 mm craniotomy was performed on the right hemisphere as described above. Mice received injection of adeno-associated virus serotype 2 (AAV2)-ChR2-mCherry, AAV2-Gria1-P2A-eGFP or AAV2-eGFP (control) into the DCN (AP = −6.4 mm; ML = −1.3 mm; DV = +2.5–3 mm from the skull surface) at a viral titer of 5 × 10^12^ vg/mL. AAV-ChR2-mCherry, AAV2-Gria1-P2A-eGFP, or AAV2-eGFP (control) (1 µL) was infused using a 26-gauge needle attached to a 10-µL Hamilton syringe at a slow rate of 0.2 µL/min (Pump11, Harvard Apparatus), and the syringe was left in place for an additional 5 min before being slowly withdrawn. Mice were then allowed to recover for 2 weeks after AAV-ChR2-mCherry or AAV2-Gria1-P2A-eGFP injection.

### Optogenetic stimulation of DCN with evoked EMG recording

For optical stimulation of DCN, optical fiber with blue laser at 473 nm (Inper Inc.) was illuminated at 50 μm above the injection site with 10 ms pulse and constant laser light power at 1 mW. Intensity output from the optical fiber was verified before and after each experiment, to be about 1 mW by a PM20A fiber power meter (Thorlabs)^109^. The optical fiber was coupled to the electrophysiology data-acquisition system (Blackrock microsystem) driven by software-generated transistor-to-transistor logic pulses, to align laser stimulation and EMG recording. DCN was stimulated with simultaneous EMG recording of gastrocnemius muscle for 30 min. An average of 25–30 EMG responses for each mouse were used for CMAP amplitudes and latency calculation^48^.

### Patient database and selection of study patients

The research protocol for this retrospective study was reviewed by the Mayo Clinic Institutional Review Board Committee and was exempt from the requirement for IRB approval (IRB decision 20-006692).

Mayo Clinic electronic medical record database EPIC was searched with Advanced Cohort Explorer (ACE) software. This program interrogates the electronic medical record in a similar fashion to an internet search. The search was conducted using the following inclusive search terms: peripheral nerve damage, peripheral neuropathy, facial nerve neuropathy, median nerve neuropathy, radial neuropathy, ulnar neuropathy, sciatic nerve neuropathy, tibial neuropathy, and peroneal neuropathy. All these search terms were then cross-referenced with a second search term which included cerebellar stroke, spinocerebellar ataxia, cerebellar ataxia, cerebellar damage, cerebellar injury, cerebellar atrophy, cerebellar degeneration, olivo-ponto-cerebellar atrophy, and multiple system atrophy. We focused further analysis on those ataxia patients and non-ataxia patients (controls) who underwent a corrective surgical procedure for CTS release or after being diagnosed with tibial neuropathy. In the final manual selection stage, only the patients with a precise date of peripheral nerve lesion diagnosis and those with at least one follow-up visit with a proper nerve conduction study (NCS) done after the surgical intervention (for CTS patients) or being diagnosed with tibial neuropathy, were selected for analysis.

For the control group, each patient from the ataxia study group was matched with at least one patient with a similar peripheral nerve injury without any cerebellar damage (non-ataxia patient). The control patients were selected manually from the group of patients obtained in the primary search through ACE. All ataxia and control patients had brain imaging (MRI or computerized tomography) performed, and they underwent identical Mayo Clinic standardized NCS protocol^110^ for CMAP amplitude and motor nerve conduction velocity measurements.

### Gene expression omnibus (GEO) database analysis

To determine the expression profiles of human GRIA1 (NM_000827) in ataxia patients, gene expression datasets from ataxia patient samples were first identified from GEO (NCBI GEO; https://www.ncbi.nlm.nih.gov/geo/), using ‘ataxia’ as a keyword. Initial screening was performed in November 2021 and identified a total of 121 datasets that were derived from human samples. Samples that were derived from the patient's peripheral blood, blood plasma, and immune cells were excluded from the gene expression analysis. We considered samples derived from ataxia patients such as the cerebellum (GSE61019), patient iPSC-derived Purkinje cell and neuron (GSE85349 and GSE96826), patient-derived iPSCs (GSE35347, GSE45030), and patient-derived fibroblasts (GSE6971, GSE27041, GSE33940, GSE104288, GSE152287, and GSE175477) and their pre-processed Series Matrix Files (expression quantification matrices) were extracted from NCBI GEO databases for subsequent analysis. The gene expression level of human GRIA1 from each sample was normalized to the average GRIA1 expression level in healthy individuals for relative log2 fold change calculation.

### Statistical analysis

Statistical analysis was performed using GraphPad Prism software (Version 8.0). For multiple comparisons in behavioral and electrophysiology study, two-way ANOVA was used followed by appropriate post hoc analysis stated in the figure legends. Unpaired Student’s *t* test was used to compare two groups. In vitro data was analyzed using one-way ANOVA with Bonferroni’s multiple comparison test. All experimental data are presented as the mean ± SEM and clinical data as mean ± SD.

### Reporting summary

Further information on research design is available in the Nature Research Reporting Summary linked to this article.

## Supplementary information

Supplementary Information

Reporting Summary

## Data Availability

All the data are available in the main text or supplementary materials. The materials are available from the corresponding author upon reasonable request. All the pre-processed microarray and RNA-seq datasets were acquired directly from Gene Expression Omnibus under accession numbers GSE6971, GSE27041, GSE33940, GSE35347, GSE45030, GSE61019, GSE85349, GSE96826, GSE104288, GSE152287, and GSE175477.

## Code availability

The MATLAB code used in the current study is available from the corresponding author upon reasonable request.
