# Supplementary material for: Cerebellar glutamatergic system impacts spontaneous motor recovery by regulating Gria1 expression
Source: NPJ Regen Med. 2022 Sep 5;7:45. doi: 10.1038/s41536-022-00243-6 (PMC9445039; doi:10.1038/s41536-022-00243-6)
Supplement: Supplementary file 1 — Supplementary Information [file 41536_2022_243_MOESM1_ESM.pdf]

Supplementary Information

Title: Cerebellar glutamatergic system impacts spontaneous motor recovery by regulating *Gria1* expression

Authors: Pallavi Asthana[1]†, Gajendra Kumar[1]†, Lukasz M. Milanowski[2,3], Ngan Pan Bennett Au[1], Siu Chung Chan[1], Jianpan Huang[4], Hemin Feng[1], Kin Ming Kwan[5], Jufang He[1], Kannie Wai Yan Chan[4,6], Zbigniew K. Wszolek[2], Chi Him Eddie Ma[1]*

† Authors contribute equally

Affiliations: [1]Department of Neuroscience, City University of Hong Kong, Tat Chee Avenue, Hong Kong SAR. [2]Department of Neurology, Mayo Clinic, Jacksonville, USA. [3]Department of Neurology, Faculty of Health Science, Medical University of Warsaw, Warsaw, Poland. [4]Department of Biomedical Engineering, City University of Hong Kong, Tat Chee Avenue, Hong Kong SAR. [5]School of Life Sciences, Center for Cell and Developmental Biology and State Key Laboratory of Agrobiotechnology, The Chinese University of Hong Kong, Shatin, Hong Kong SAR. [6]Russell H. Morgan Department of Radiology and Radiological Science, Johns Hopkins University School of Medicine, Baltimore, USA.

*Corresponding author
Dr. Chi Him Eddie Ma
Department of Neuroscience, City University of Hong Kong, Tat Chee Avenue, Hong Kong SAR
Email: eddiema@cityu.edu.hk
Tel: (852) 3442 9328
Fax: (852) 3442 0549

Numbers of Supplementary Figures: 11

Numbers of Supplementary Tables: 3

Supplemenatary Figure Legends

Supplementary Figure 1. The rate of *in vivo* axon regeneration of sensory axons is unaffected in ataxia mice. (a) Sciatic nerve pinch test was performed after 72 hours of sciatic nerve crush to assess the extent of *in vivo* axon regeneration in control and ataxia mice. Distal extent of axonal regrowth was comparable between control (2.91 mm) and ataxia mice (2.87 mm). Values represent the Mean ± SEM (n = 5 mice per group); unpaired Student's *t*-test. ns; non-significant.

Supplementary Figure 2. Intrinsic growth capacity of injured dorsal root ganglion (DRG) neurons remains unaffected in ataxia mice. (a) Prior to DRG culture animals had been subjected to preconditioning nerve injury (sciatic nerve transection). **(b)** Representative immunofluorescence microphotographs of DRG neurons immunostained with anti-β-tubulin III antibody, showing longer neurite of injured neurons as compared to uninjured neurons from both control and ataxia mice. Neurites outgrowth of injured DRG neurons from ataxia mice following preconditioning was comparable to that of the injured neurons from control group. Scale bar: 100µm. **(c)** Preconditioned neurons from control and ataxia mice showed 1.68- to 1.7-fold increase in neurite length (530 ± 18.89 µm in control and 549 ± 16.29 µm in ataxia) when compared with uninjured neurons (305 ± 9.43 µm in control and 318 ± 9.99 µm in ataxia). Injured side is referred as ipsilateral whereas uninjured side as contralateral. Values represent the Mean ± SEM from three independent experiments performed in triplicate (180 – 271 neurons were measured per condition); *** $P < 0.001$, One-way ANOVA multiple comparison test.

Supplementary Figure 3. Ataxia mice show delayed motor function recovery within the critical period of muscle denervation. (a) Schematic diagram illustrating the experimental paradigm for the animal model of 16 days of muscle denervation. **(b)** Toe spread response was fully recovered in control mice and was significantly delayed in ataxia mice. **(c)** Hindlimb grip strength remained low in ataxia mice and was unable to restore to baselines, while control mice regained full muscle strength in about 3 weeks. **(d-e)** Functional neuromuscular junction reinnervation was measured by weekly electromyography recording of proximal (gastrocnemius, GCM) and distal (interosseous) plantar muscles for 2 months. Compound muscle action potential (CMAP) amplitudes of ataxia mice were significantly lower than control mice throughout the assessment period. n = 9-10 mice per group. Values represents the Mean ± SEM; two-way ANOVA followed by *post hoc* Bonferroni test; * $P < 0.05$; ** $P < 0.01$; *** $P < 0.001$ when compared with control group. Detailed statistics analyses comparing CMAP amplitude data points across time within groups are available in the Supplementary Table 3.

Supplementary Figure 4. Ataxia mice shortens the critical period to 22 days. (a) Schematic diagram illustrating the experimental paradigm for the animal model of 22 days of muscle denervation. **(b)** Ataxia mice did not show minimal toe spread reflex after the end of a 2-month assessment period. Control mice showed clear toe spreading reflex and complete recovery within 1 month. **(c-e)** Hindlimb grip strength and Compound muscle action potential (CMAP) amplitudes of ataxia mice remained significantly below the baseline values throughout the assessment period, indicating irreversible motor deficits. n = 8-12 mice per group. Values represent the Mean ± SEM; two-way ANOVA followed by *post hoc* Bonferroni test; * $P < 0.05$; ** $P < 0.01$; *** $P < 0.001$ when compared with control group. Detailed statistics analyses comparing CMAP amplitude data points across time within groups are available in the Supplementary Table 3.

Supplementary Figure 5. Sensory function recovery and motor coordination in ataxia mice remains unaffected across different muscle denervation models. (a) Sensory function recovery measured by pinprick assay and was complete within three weeks after 16, 22 and 25 days of muscle denervation in control and ataxia mice. No observable differences between the groups. **(b)** Motor coordination was impaired before the sciatic nerve crush in ataxia mice assessed by accelerated rotarod tasks. Latency to fall from the rotarod was further reduced in ataxia mice after injury across different muscle denervation models. Motor coordination of control mice returned to pre-injury baseline values. n = 8-12 mice per group. Values represent the Mean ± SEM; two-way ANOVA followed by *post hoc* Bonferroni test; *** $P < 0.001$ when compared with control group.

Supplementary Figure 6. Extensive spatial spread and high expression levels of AAV2-ChR2-mCherry in deep cerebellar nuclei (DCN). Mice received injection of adeno-associated virus serotype 2 (AAV2)-channel rhodopsin-2 (ChR2)-mCherry into the DCN. Thirty-micron-thick sagittal cerebellum sections were imaged to cover the maximum area of cerebellum for identification of the DCN based on the surrounding folia structure 2 weeks after the injections. Robust transduction efficacy of AAV2-ChR2-mCherry was observed in the DCN. No mCherry expression was detected in the Purkinje cell layer and in the white matter region. The corresponding sagittal section of the mouse brain in the Allen Mouse Brain Atlas (bregma: -6.4mm) (left panel). Scale bar: 500 µm.

Supplementary Figure 7. Ataxia mice exhibits increased deep cerebellar nuclei (DCN) hyperexcitability after injury. Mice were implanted with 16-channel multielectrode arrays (MEAs) and DCN recording. Data were analysed using Spike2 software, Blackrock offline spike sorting (BOSS) software for neurospike sorting. **(a)** Representative baseline and post-injury DCN neural firing patterns after a single sciatic nerve crush (SNC) were shown. **(b-c)** DCN neural firing rate and number of bursts in ataxia mice was increased gradually at 2-week post-SNC onwards, but remained significantly lower than the controls. **(d)** Representative interspike intervals (ISI) distribution heat maps were shown. **(e)** Average ISI of ataxia mice were significantly lower than control mice, indicating persistent aberrant neural firing occurred in ataxia mice. n = 6-8 mice per group. Values represent the Mean ± SEM; two-way ANOVA followed by *post hoc* Bonferroni test; *** $P < 0.001$ when compared with control group.

Supplementary Figure 8. Deep cerebellar nuclei (DCN) stimulation restores cerebellar circuitry and motor function after peripheral nerve injury. (a) Schematic diagram illustrating the experimental paradigm for a single sciatic nerve crush (SNC) and DCN deep brain stimulation (DBS) treatment. Daily 1-h DCN-DBS was started after the SNC for 21 consecutive days. **(b-c)** DCN neural firing rate and number of bursts were comparable between control and ataxia mice. Representative DCN neural firing patterns were shown in the left panel of b. **(d)** Average interspike intervals (ISI) of ataxia mice were increased gradually and comparable to control from week 2 post-SNC onwards. Representative ISI distribution heat maps were shown in the left panel. **(e-f)** Daily DCN-DBS restored spontaneous motor recovery in ataxia mice to a similar extent as control mice. **(g-h)** Ataxia mice with DCN-DBS recovered compound muscle action potential (CMAP) amplitudes from proximal and distal muscles gradually which were comparable to the control levels. **(i)** Sensory function recovery was fully recovered without any delays in all the groups. n = 6-8 mice per group for DCN neurospike firing analysis (b-d), neurobehavioral experiments (e-f, i), and electromyography recording studies (g-h). Values represent the Mean ± SEM; two-way ANOVA followed by *post hoc* Bonferroni test; * $P < 0.05$; ** $P < 0.01$; *** $P < 0.001$. In e- f, * $P < 0.05$ when compared with the ataxia mouse group

without DBS. ns; non-significant. Detailed statistics analyses comparing CMAP amplitude data points across time within groups are available in the Supplementary Table 3.

Supplementary Figure 9. Baclofen treatment restores neural firing pattern in ataxia mice. (a) Schematic diagram illustrating the experimental paradigm for a single sciatic nerve crush and baclofen treatment. **(b)** Average neural firing rate **(c)** interspike interval (ISI) and **(d)** number of bursts in baclofen-treated ataxia mice were restored to near control values. Representative DCN neural firing patterns and ISI distribution heat maps were shown in b-c, respectively. n = 5-6 mice per group. Values represent the Mean ± SEM; two-way ANOVA followed by *post hoc* Bonferroni test; *** $P < 0.001$ when compared with the control mouse group treated with baclofen.

Supplementary Figure 10. Ataxia mice fail to upregulate mRNA expression of Gria1 in the cerebellum at day 7 post-sciatic nerve crush (SNC). (a) qPCR analysis on the mRNA expression levels of AMPA subtype of ionotropic glutamate receptors revealed that the mRNA expression of *Gria1* was remained unchanged in ataxia mice, while the *Gria1* expression was upregulated in control mice after SNC. **(b)** qPCR analysis on the mRNA expression levels of NMDA subtype of ionotropic receptors showed increased expression of *Grin2b* and *Grin2c* in both control and ataxia mice. **(c)** DNQX treatment did not affect the sensory functional recovery after SNC. **(d)** The open reading frame of mouse *Gria1* was sub-cloned into a pCF-CMV-P2A-eGFP vector and packaged into adeno-associated virus serotype 2 (AAV2) for intracerebroventricular injection. **(e)** A significant upregulation of *Gria1* was detected in the cerebellum of ataxia mice treated with AAV-Gria1 at 1.5 months after intracerebroventricular injection of AAV-Gria1. **(f)** No difference in sensory function recovery was detected in these three mouse groups after SNC. Values represent the Mean ± SEM of triplicate in (a, b and e); n = 9-10 per group in c; n = 7 for ataxia + AAV-eGFP group; n = 9 for control + AAV-eGFP and ataxia + AAV-Gria1 groups in f; * $P < 0.05$; compared with uninjured naïve control mice in (a, b and e), or vehicle-treated controls in (c), or control mice with AAV-eGFP in (f); # $P < 0.05$, compared with ataxia mice in (a), or in ataxia mice with AAV-eGFP in (f), one-way ANONA followed by *post hoc* Bonferroni test in (a, b and e); two-way ANOVA with repeated measures followed by *post hoc* Bonferroni test in (c and f).

Supplementary Figure 11. Optimizing electrical stimulation parameters for deep cerebellar nuclei (DCN), motor cortex and spinal cord with evoked electromyography (EMG) recording. The optimal stimulation current was determined as the stimuli which induced maximal EMG response before reaching plateau. The input/output protocol was repeated in each individual mouse that subjected to electrical stimulation to ensure reproducibility. Optimised stimulation parameters (100μA current, 100μs pulse width, 1Hz frequency) was used for electrical stimulation of DCN, motor cortex and spinal cord with evoked EMG recording. A set of five stimuli was delivered for each stimulation current at 1 Hz and increased by increments of 25 μA ranging from 25 to 200 μA. Values represent the Mean ± SEM (n =3 mice per group).

Supplementary Table 1. Sequences of the primers used for qPCR.

Supplementary Table 2. Statistics analyses comparing data points across time within groups for CMAP amplitudes data in the main figures.

Supplementary Table 3. Statistics analyses comparing data points across time within groups for CMAP amplitudes data in the supplementary figures.

Supplementary Figure 1

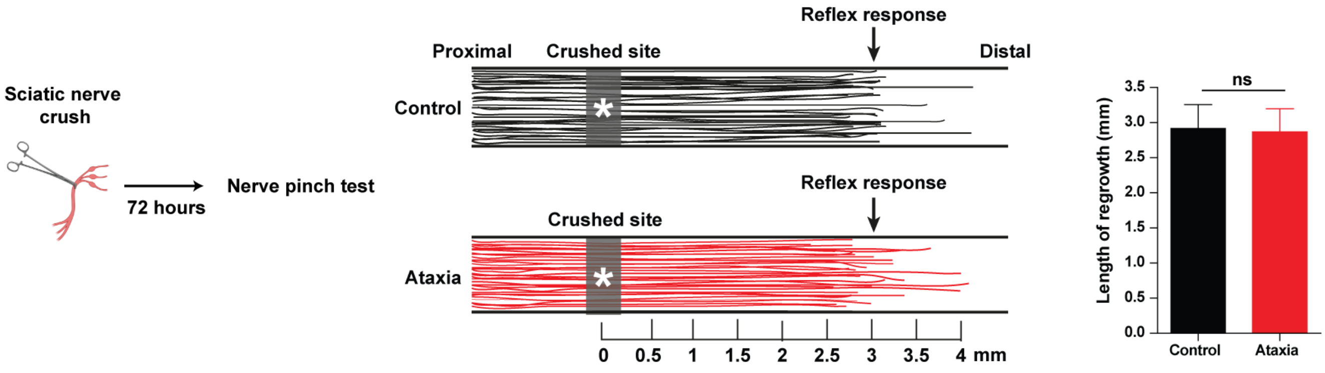

Supplementary Figure 2

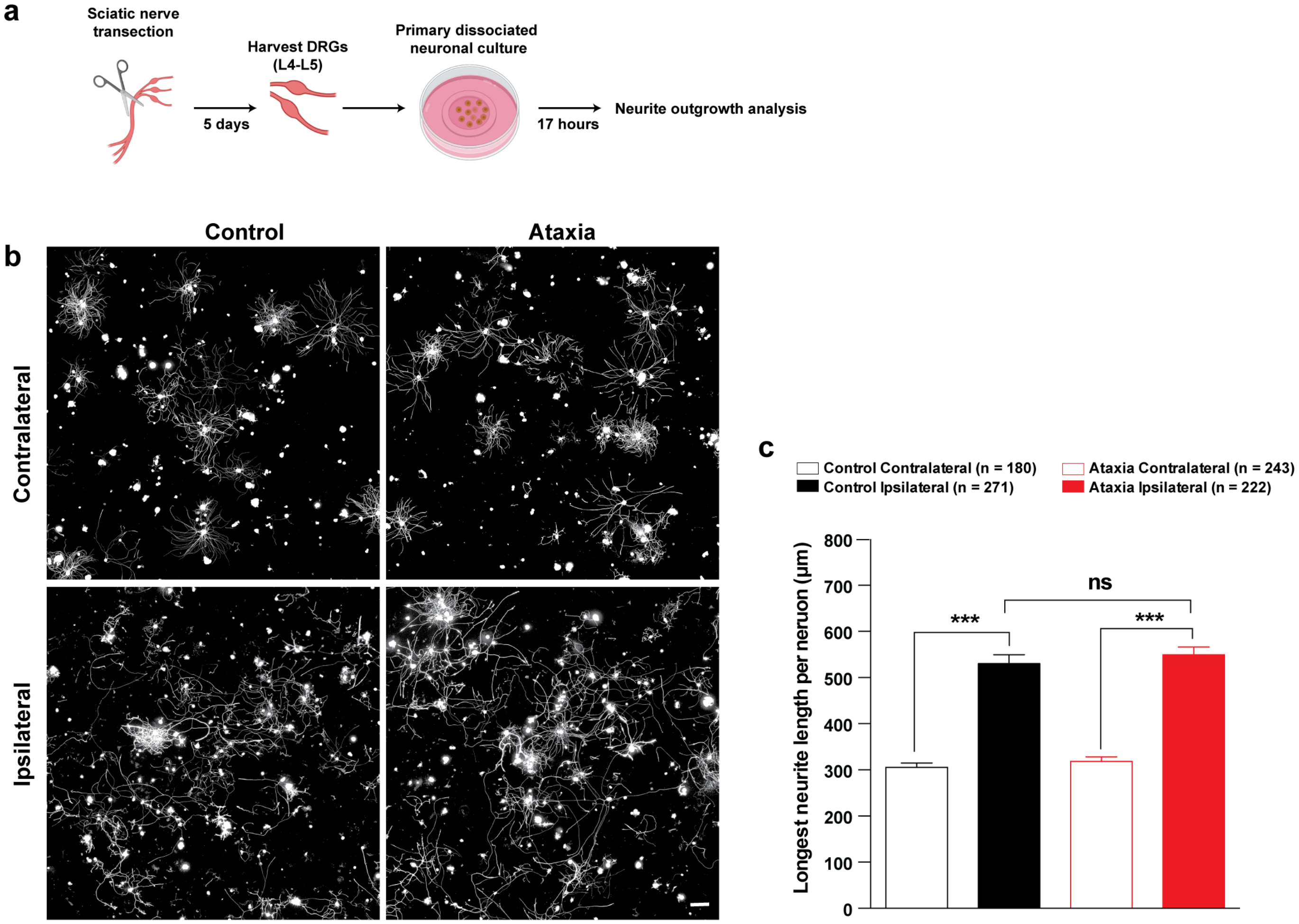

Supplementary Figure 3

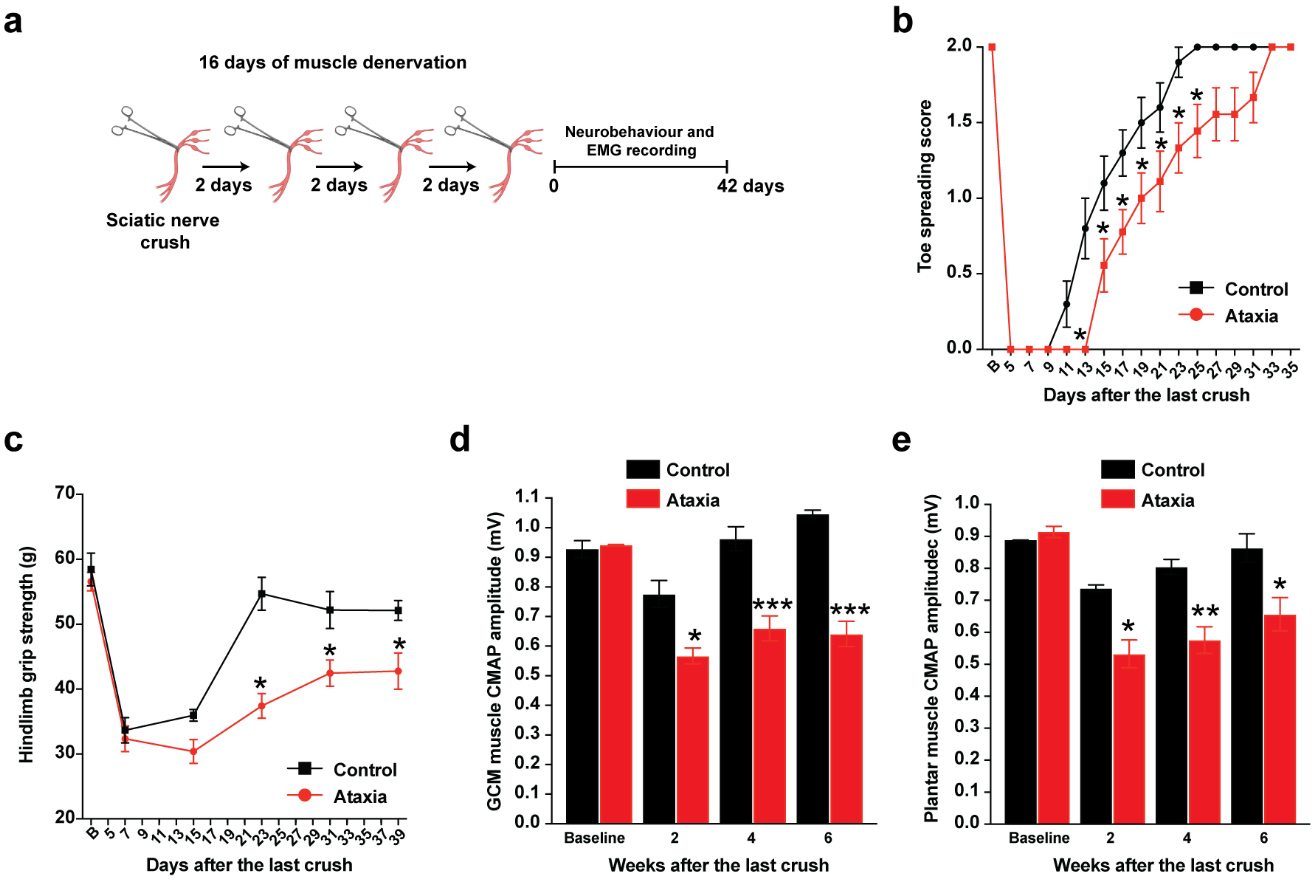

Supplementary Figure 4

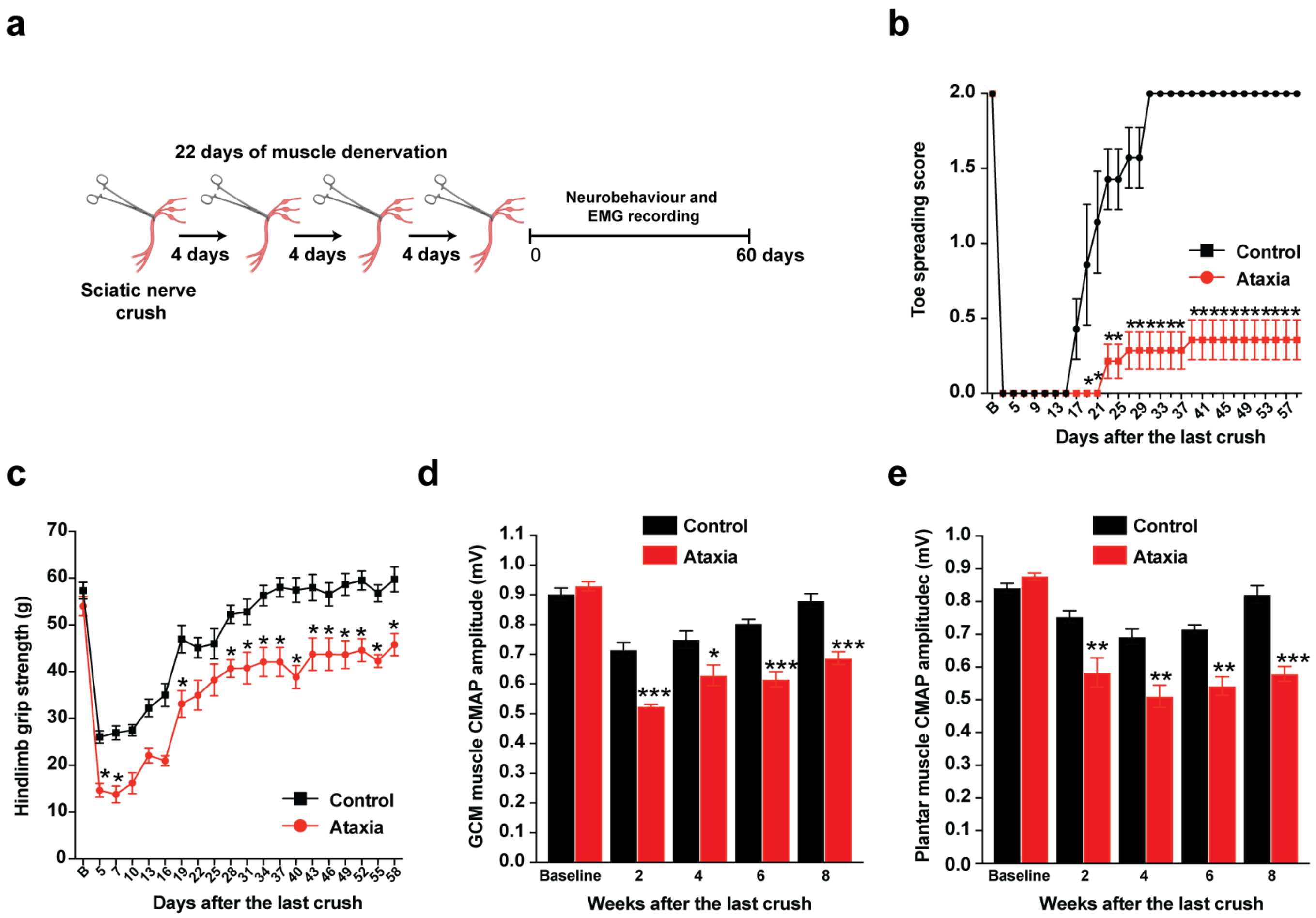

Supplementary Figure 5

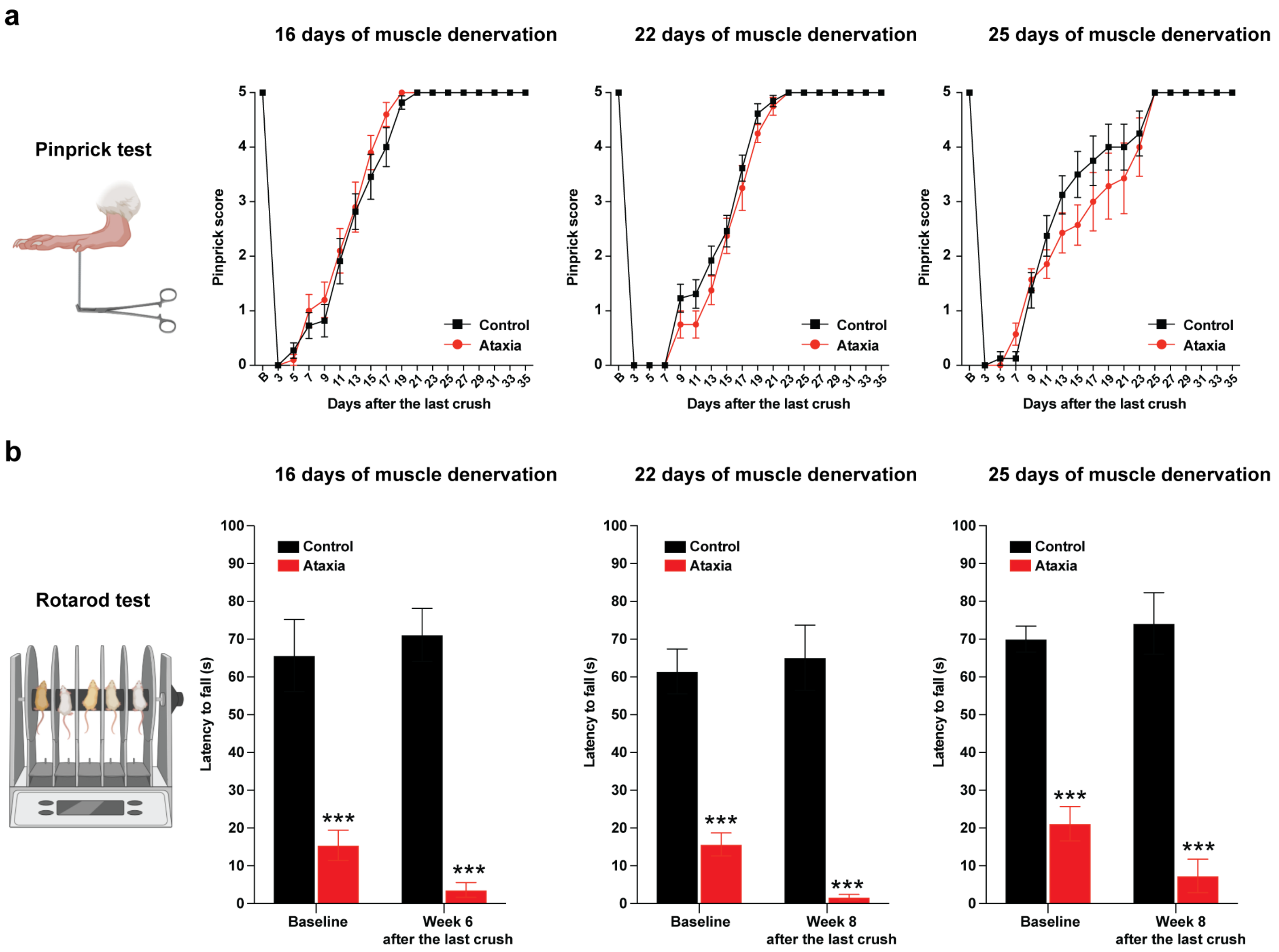

Supplementary Figure 6

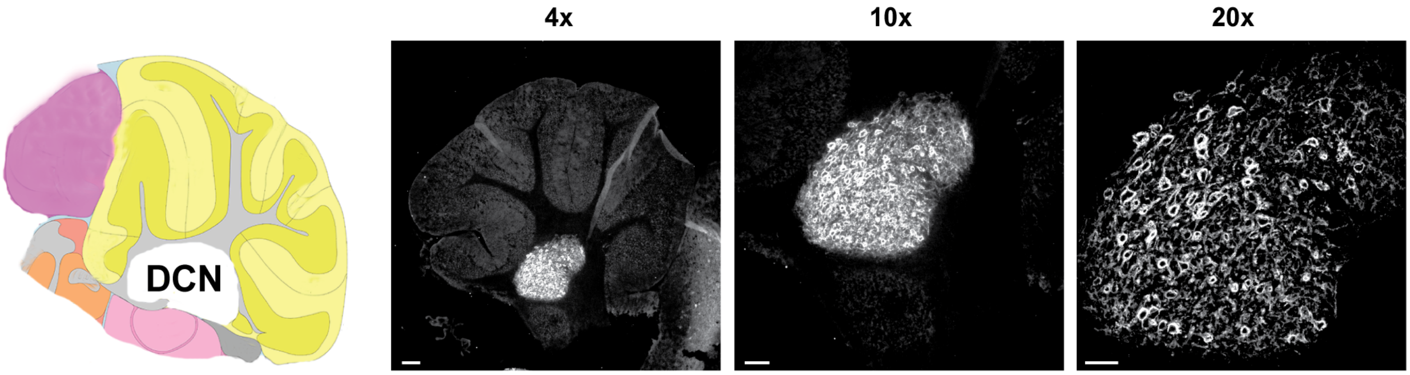

Supplementary Figure 7

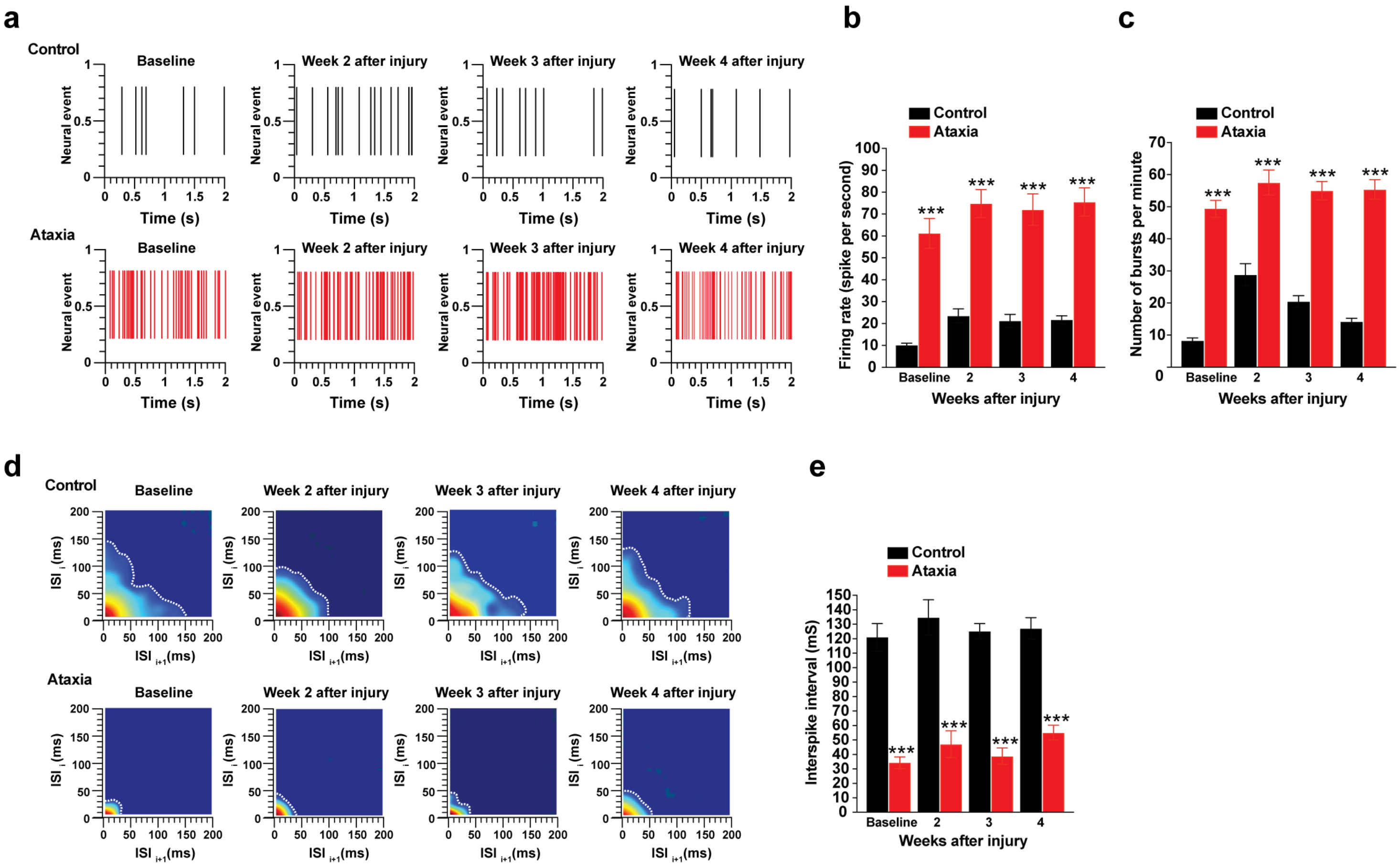

Supplementary Figure 8

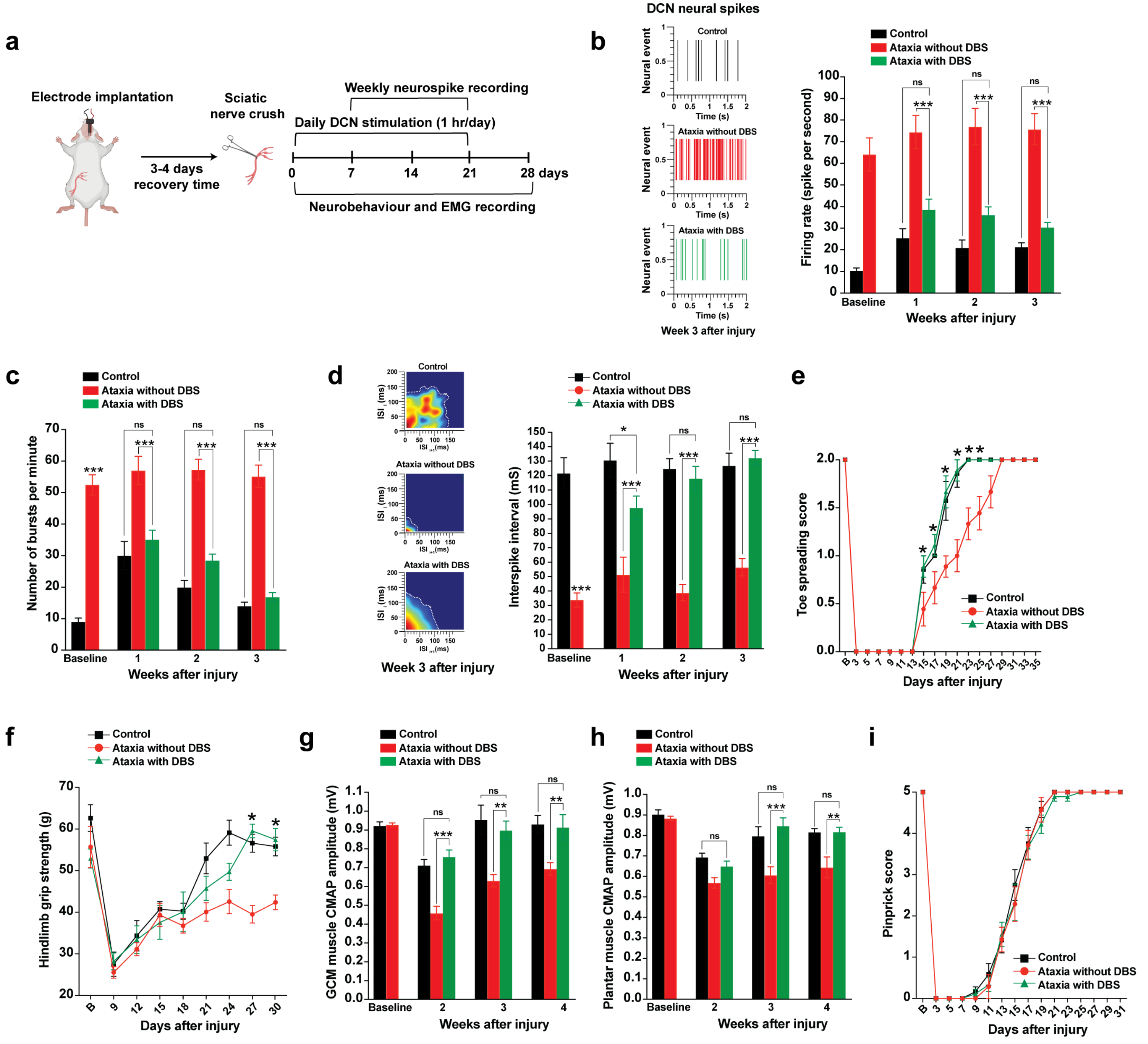

Supplementary Figure 9

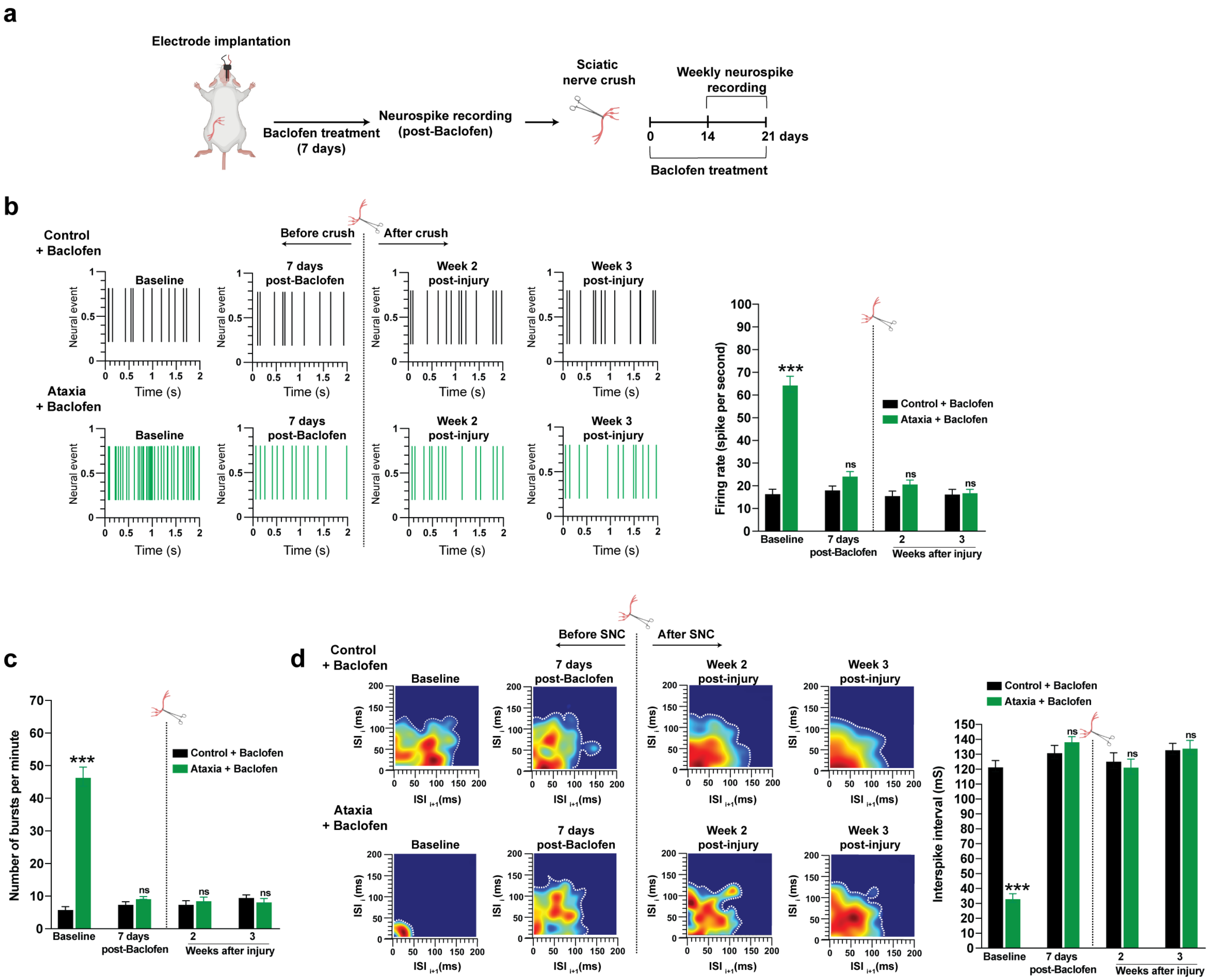

Supplementary Figure 10

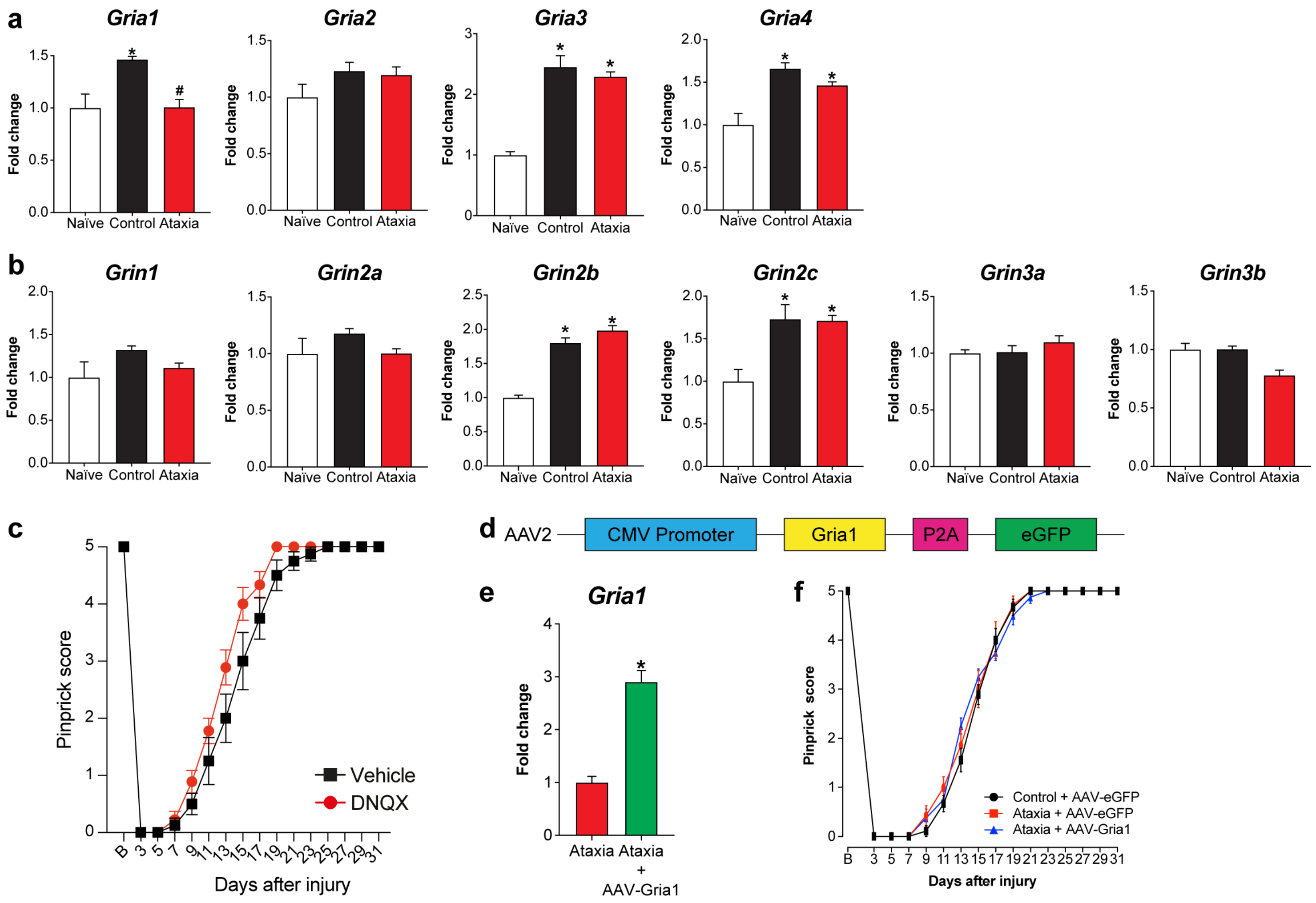

Supplementary Figure 11

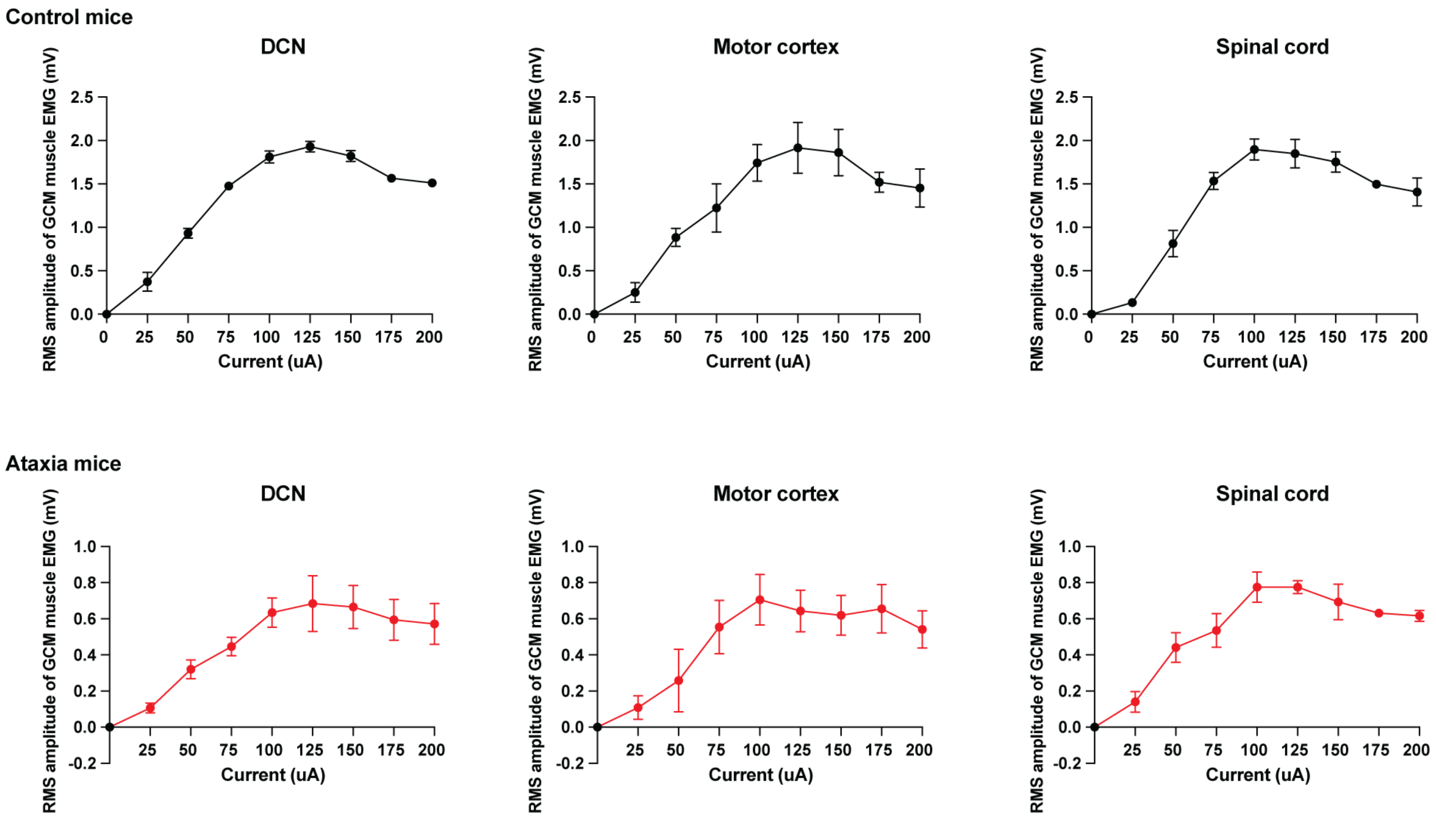

Supplementary Table 1

Gene Name	Sequence	
Gria1	Forward	GGTGCCAATTTCCCCAACAA
	Reverse	GCTGTCGCTGATGTTCACAA
Gria2	Forward	GGTTGTCACCACAATATTGGAATC
	Reverse	GAGAAGTCAATCACCTCTTCTCTC
Gria3	Forward	AGGGGTACGGTTAAGATCCCT
	Reverse	AGGTCATTGCACCATCAGCA
Gria4	Forward	TGACATTTCACCCAGATCCC
	Reverse	TTCTGCACTTTCTATGGGGG
Grin1	Forward	CCTATGACAACAAGCGCGG
	Reverse	AGTCCGATGATGCCGTCAG
Grin2a	Forward	AGCTTGAAAACTGGGAAGTTGG
	Reverse	GTGGTGGCAAAGATGTACCC
Grin2b	Forward	CCTCCTGTGTGAGAGGAAAGAA
	Reverse	GTGGTCATTCCCAAAGCGTC
Grin2c	Forward	ACAAGCCTGAAGATGGGGAAG
	Reverse	TTTGCCAGACCCAATGGTGA
Grin3a	Forward	ACGTGTGGAAAAGAGGTCCA
	Reverse	AGGGATGTCCTGATGGGTCT
Grin3b	Forward	CACACGAGCCAGAAGATCCA
	Reverse	TGCAGGTTGCTCCTCCTTG

Supplementary Table 2

Sciatic nerve crush					
Statistical analysis for Figure 1		Week2	Week3	Week4	
Figure 1f (GCM)	Control baseline vs Control post-crush	****; $P < 0.0001$	ns	ns	
	Ataxia baseline vs Ataxia post-crush	****; $P < 0.0001$	****; $P < 0.0001$	****; $P < 0.0001$	
	Control baseline vs Ataxia post-crush	****; $P < 0.0001$	****; $P < 0.0001$	****; $P < 0.0001$	
Figure 1g (LPM)	Control baseline vs Control post-crush	**; $P = 0.0032$	ns	ns	
	Ataxia baseline vs Ataxia post-crush	****; $P < 0.0001$	****; $P < 0.0001$	****; $P < 0.0001$	
	Control baseline vs Ataxia post-crush	****; $P < 0.0001$	****; $P < 0.0001$	****; $P < 0.0001$	
25 days of denervation		Week2	Week4	Week6	Week8
Figure 1l (GCM)	Control baseline vs Control post-crush	*; $P = 0.0223$	ns	ns	ns
	Ataxia baseline vs Ataxia post-crush	****; $P < 0.0001$	***; $P = 0.0003$	*; $P = 0.0116$	*; $P = 0.0180$
	Control baseline vs Ataxia post-crush	****; $P < 0.0001$	***; $P = 0.0004$	*; $P = 0.0188$	*; $P = 0.0281$
Figure 1m (LPM)	Control baseline vs Control post-crush	***; $P = 0.0002$	ns	ns	ns
	Ataxia baseline vs Ataxia post-crush	****; $P < 0.0001$	****; $P < 0.0001$	****; $P < 0.0001$	****; $P < 0.0001$
	Control baseline vs Ataxia post-crush	****; $P < 0.0001$	****; $P < 0.0001$	****; $P < 0.0001$	****; $P < 0.0001$

DCN lesion and Hippocampus lesion			
Statistical analysis for Figure 2		Week2	Week4
Figure 2e (GCM)	Sham baseline vs Sham post-crush	*; $P = 0.0202$	ns
	DCN Lesion baseline vs DCN Lesion post-crush	****; $P < 0.0001$	****; $P < 0.0001$
	Sham baseline vs DCN Lesion post-crush	****; $P < 0.0001$	***; $P = 0.0003$
Figure 2f (LPM)	Sham baseline vs Sham post-crush	-	ns
	DCN Lesion baseline vs DCN Lesion post-crush	-	****; $P < 0.0001$
	Sham baseline vs DCN Lesion post-crush	-	****; $P < 0.0001$
Figure 2n (GCM)	Sham baseline vs Sham post-crush	ns	ns
	Hippocampus Lesion baseline vs Hippocampus Lesion post-crush	**; $P = 0.0021$	ns
	Sham baseline vs Hippocampus Lesion post-crush	**; $P = 0.0024$	ns
Figure 2o (LPM)	Sham baseline vs Sham post-crush	-	ns
	Hippocampus Lesion baseline vs Hippocampus Lesion post-crush	-	ns
	Sham baseline vs Hippocampus Lesion post-crush	-	ns

22 days of muscle denervation and DCN stimulation					
Statistical analysis for Figure 4		Week2	Week4	Week6	Week8
Figure 4e (GCM)	Control baseline vs Control post-crush	****; $P < 0.0001$	ns	ns	ns
	Ataxia without DBS baseline vs Ataxia without DBS post-crush	****; $P < 0.0001$	****; $P < 0.0001$	****; $P < 0.0001$	****; $P < 0.0001$
	Control baseline vs Ataxia without DBS post-crush	****; $P < 0.0001$	****; $P < 0.0001$	**; $P = 0.0013$	***; $P = 0.0002$
	Control baseline vs Ataxia with DBS post-crush	*; $P = 0.0184$	ns	ns	ns
Figure 4f (LPM)	Control baseline vs Control post-crush	ns	ns	ns	ns
	Ataxia without DBS baseline vs Ataxia without DBS post-crush	****; $P < 0.0001$	****; $P < 0.0001$	****; $P < 0.0001$	****; $P < 0.0001$
	Control baseline vs Ataxia without DBS post-crush	****; $P < 0.0001$	****; $P < 0.0001$	****; $P < 0.0001$	***; $P = 0.0003$
	Control baseline vs Ataxia with DBS post-crush	*; $P = 0.0150$	ns	ns	ns

Sciatic nerve crush and Baclofen				
Statistical analysis for Figure 5		Week2	Week3	Week4
Figure 5f (GCM)	Control + Baclofen baseline vs Control + Baclofen post-crush	****; $P < 0.0001$	ns	ns
	Ataxia + Baclofen baseline vs Ataxia + Baclofen post-crush	****; $P < 0.0001$	**; $P = 0.0020$	ns
	Control + Baclofen baseline vs Ataxia + Baclofen post-crush	****; $P < 0.0001$	**; $P = 0.0034$	ns
Figure 5f (LPM)	Control + Baclofen baseline vs Control + Baclofen post-crush	****; $P < 0.0001$	*; $P = 0.0117$	ns
	Ataxia + Baclofen baseline vs Ataxia + Baclofen post-crush	****; $P < 0.0001$	****; $P < 0.0001$	ns
	Control + Baclofen baseline vs Ataxia + Baclofen post-crush	****; $P < 0.0001$	****; $P < 0.0001$	ns

Sciatic nerve crush and DNQX/AAV-Gria1 Overexpression				
Statistical analysis for Figure 6		Week2	Week3	Week4
Figure 6e (GCM)	Vehicle baseline vs Vehicle post-crush	****; $P < 0.0001$	**; $P = 0.0029$	ns
	DNQX baseline vs DNQX post-crush	****; $P < 0.0001$	****; $P < 0.0001$	*; $P = 0.011$
	Vehicle baseline vs DNQX post-crush	****; $P < 0.0001$	**; $P = 0.0014$	ns
Figure 6f (LPM)	Vehicle baseline vs Vehicle post-crush	**; $P = 0.0016$	**; $P = 0.0099$	ns
	DNQX baseline vs DNQX post-crush	****; $P < 0.0001$	****; $P < 0.0001$	ns; $P = 0.6931$
	Vehicle baseline vs DNQX post-crush	****; $P < 0.0001$	****; $P < 0.0001$	ns; $P = 0.2016$
Figure 6j (GCM)	Control + AAV-eGFP baseline vs Control + AAV-eGFP post-crush	***; $P = 0.0009$	**; $P = 0.001$	ns
	Ataxia + AAV-eGFP baseline vs Ataxia + AAV-eGFP post-crush	****; $P < 0.0001$	****; $P < 0.0001$	****; $P < 0.0001$
	Ataxia + AAV-Gria1 baseline vs Ataxia + AAV-Gria1 post-crush	****; $P < 0.0001$	****; $P < 0.0001$	ns
Figure 6k (LPM)	Control + AAV-eGFP baseline vs Control + AAV-eGFP post-crush	ns; $P = 0.2407$	ns	ns
	Ataxia + AAV-eGFP baseline vs Ataxia + AAV-eGFP post-crush	**; $P = 0.0034$	**; $P = 0.0021$	*; $P = 0.0267$
	Ataxia + AAV-Gria1 baseline vs Ataxia + AAV-Gria1 post-crush	***; $P = 0.0001$	ns	ns

Supplementary Table 3

16 days of muscle denervation				
**Statistical analysis for Supplementary Figure 3**		**Week2**	**Week4**	**Week6**
**Supplementary Figure 3d (GCM)**	Control baseline vs Control post-crush	ns	ns	ns
	Ataxia baseline vs Ataxia post-crush	**; P = 0.0019	*; P = 0.0227	*; P = 0.0151
	Control baseline vs Ataxia post-crush	**; P = 0.0029	*; P = 0.0356	*; P = 0.0235
**Supplementary Figure 3e (LPM)**	Control baseline vs Control post-crush	ns	ns	ns
	Ataxia baseline vs Ataxia post-crush	***; P = 0.0004	***; P = 0.0020	*; P = 0.0302
	Control baseline vs Ataxia post-crush	**; P = 0.0011	**; P = 0.0051	ns; P = 0.0765

22 days of muscle denervation					
**Statistical analysis for Supplementary Figure 4**		**Week2**	**Week4**	**Week6**	**Week8**
**Supplementary Figure 4d (GCM)**	Control baseline vs Control post-crush	*; P = 0.0167	ns	ns	ns
	Ataxia baseline vs Ataxia post-crush	****; P < 0.0001	****; P < 0.0001	****; P < 0.0001	****; P < 0.0001
	Control baseline vs Ataxia post-crush	****; P < 0.0001	****; P < 0.0001	****; P < 0.0001	**; P = 0.0014
**Supplementary Figure 4e (LPM)**	Control baseline vs Control post-crush	ns	ns	ns	ns
	Ataxia baseline vs Ataxia post-crush	****; P < 0.0001	****; P < 0.0001	****; P < 0.0001	****; P < 0.0001
	Control baseline vs Ataxia post-crush	****; P < 0.0001	****; P < 0.0001	****; P < 0.0001	****; P < 0.0001

Sciatic nerve crush and DCN stimulation				
**Statistical analysis for Supplementary Figure 8**		**Week2**	**Week3**	**Week4**
**Supplementary Figure 8g (GCM)**	Control baseline vs Control post-crush	ns	ns	ns
	Ataxia without DBS baseline vs Ataxia without DBS post-crush	****; P < 0.0001	**; P = 0.0045	*; P = 0.0472
	Control baseline vs Ataxia without DBS post-crush	****; P < 0.0001	**; P = 0.0057	ns; p=0.0601
	Control baseline vs Ataxia with DBS post-crush	ns	ns	ns
**Supplementary Figure 8h (LPM)**	Control baseline vs Control post-crush	***; P = 0.0024	ns	ns
	Ataxia without DBS baseline vs Ataxia without DBS post-crush	****; P < 0.0001	****; P < 0.0001	***; P = 0.0002
	Control baseline vs Ataxia without DBS post-crush	****; P < 0.0001	****; P < 0.0001	***; P = 0.0002
	Control baseline vs Ataxia with DBS post-crush	***; P = 0.0001	ns	ns
