# Supplementary material for: Cerebellar glutamatergic system impacts spontaneous motor recovery by regulating Gria1 expression
Source: NPJ Regen Med. 2022 Sep 5;7:45. doi: 10.1038/s41536-022-00243-6 (PMC9445039; doi:10.1038/s41536-022-00243-6)
Supplement: Supplementary file 2 — Reporting Summary [file 41536_2022_243_MOESM2_ESM.pdf]

nature portfolio

Corresponding author(s): Dr. Chi Him Eddie Ma

Last updated by author(s): May 5, 2022

Reporting Summary

Nature Portfolio wishes to improve the reproducibility of the work that we publish. This form provides structure for consistency and transparency in reporting. For further information on Nature Portfolio policies, see our Editorial Policies and the Editorial Policy Checklist.

Statistics

For all statistical analyses, confirm that the following items are present in the figure legend, table legend, main text, or Methods section.

n/a	Confirmed	
☐	☒	The exact sample size (*n*) for each experimental group/condition, given as a discrete number and unit of measurement
☐	☒	A statement on whether measurements were taken from distinct samples or whether the same sample was measured repeatedly
☐	☒	The statistical test(s) used AND whether they are one- or two-sided<br>*Only common tests should be described solely by name; describe more complex techniques in the Methods section.*
☒	☐	A description of all covariates tested
☒	☐	A description of any assumptions or corrections, such as tests of normality and adjustment for multiple comparisons
☐	☒	A full description of the statistical parameters including central tendency (e.g. means) or other basic estimates (e.g. regression coefficient) AND variation (e.g. standard deviation) or associated estimates of uncertainty (e.g. confidence intervals)
☒	☐	For null hypothesis testing, the test statistic (e.g. *F*, *t*, *r*) with confidence intervals, effect sizes, degrees of freedom and *P* value noted<br>*Give P values as exact values whenever suitable.*
☒	☐	For Bayesian analysis, information on the choice of priors and Markov chain Monte Carlo settings
☒	☐	For hierarchical and complex designs, identification of the appropriate level for tests and full reporting of outcomes
☒	☐	Estimates of effect sizes (e.g. Cohen's *d*, Pearson's *r*), indicating how they were calculated

Our web collection on statistics for biologists contains articles on many of the points above.

Software and code

Policy information about availability of computer code

Data collection	NIS-Elements (Nikon) or Zen Black (Carl Zeiss) software was used for image acquisition.<br>Blackrock Microsystem was used for EMG and LFP recordings.<br>QuickTime (Apple) Player was used for video recording during PLR test.
Data analysis	WIS-NeuroMath software was used for neurite outgrowth assay.<br>Spike2 software and a customized MATLAB code were used for EMG and LFP recording analysis.<br>ImageJ software was used for for axon/NMJ quantification, RGC counting, and fluorescence intensity measurement.<br><br>R software was used to process the microarray dataset using the 'expresso' function and the MAS5 method, and WGCNA analysis. Database for Annotation, Visualization and Integrated Discovery (DAVID) platform was used for gene ontology (GO) and pathway enrichment analysis. Connectivity map database (build 02) was used for small molecule screening.<br><br>Statistical analysis was performed and all graphs were generated using GraphPad Prism 9.

For manuscripts utilizing custom algorithms or software that are central to the research but not yet described in published literature, software must be made available to editors and reviewers. We strongly encourage code deposition in a community repository (e.g. GitHub). See the Nature Portfolio guidelines for submitting code & software for further information.

Data

Policy information about availability of data

All manuscripts must include a data availability statement. This statement should provide the following information, where applicable:

- Accession codes, unique identifiers, or web links for publicly available datasets
- A description of any restrictions on data availability
- For clinical datasets or third party data, please ensure that the statement adheres to our policy

Microarray data are deposited in Gene Expression Omnibus under accession number GSE200112.

Field-specific reporting

Please select the one below that is the best fit for your research. If you are not sure, read the appropriate sections before making your selection.

☒ Life sciences ☐ Behavioural & social sciences ☐ Ecological, evolutionary & environmental sciences

For a reference copy of the document with all sections, see nature.com/documents/nr-reporting-summary-flat.pdf

Life sciences study design

All studies must disclose on these points even when the disclosure is negative.

Sample size	No statistical methods were used to predetermine sample sizes, but sample sizes used are similar to sample sizes generally employed in the field for similar experiments.
Data exclusions	No data was excluded.
Replication	For in vitro DRG cultures and ex vivo DRG explant cultures, 3 separated experiments were performed to assess the neurite outgrowth.<br>For all animal behavioral studies, 5-13 mice were used to assess sensory and motor functional recovery after sciatic nerve crush injury, or PLR test after optic nerve crush injury.<br>For immunohistochemistry, at least 3-6 mice were used in each experimental condition.<br>For sciatic nerve pinch test, at least 5-6 mice were used to determine the distal extent of sensory axonal regrowth.<br>For microarray and qPCR analysis, RNA samples was pooled from 3 animals and repeated for at least 3 times.<br>For visualize regenerating axons at multiple subcortical visual targets, at least 3-4 mice were used in each treatment group to determine the average fluorescence intensity of CTB-positive axons in each visual target.
Randomization	Adult male mice (8-12 weeks old) were randomly assigned into each experimental group for further treatments and were thereafter evaluated blind to their experimental condition.
Blinding	All behavioral assessment, quantifications and data analysis were performed by an experimenter blinded to treatment.

Reporting for specific materials, systems and methods

We require information from authors about some types of materials, experimental systems and methods used in many studies. Here, indicate whether each material, system or method listed is relevant to your study. If you are not sure if a list item applies to your research, read the appropriate section before selecting a response.

Materials & experimental systems

n/a	Involved in the study	
☐	☒	Antibodies
☒	☐	Eukaryotic cell lines
☒	☐	Palaeontology and archaeology
☐	☒	Animals and other organisms
☒	☐	Human research participants
☒	☐	Clinical data
☒	☐	Dual use research of concern

Methods

n/a	Involved in the study	
☒	☐	ChIP-seq
☒	☐	Flow cytometry
☒	☐	MRI-based neuroimaging

Antibodies

Antibodies used	Mouse anti-βIII-tubulin (1:800; Sigma-Aldrich; T8660)<br>Rabbit anti-GAP43 (1:1,000; Millipore; AB5220)<br>Rabbit anti-RBPMS (1:500; Abcam; ab194213)<br>Chicken anti-NF-200 (1:1,500; Millipore; AB5539)

	α-Bungarotoxin, Alexa Fluor 488 conjugate (1:200; Molecular Probes; B13422)<br>Rabbit anti-CD68 (1:500; Abcam; ab125212)
Validation	Mouse anti-βIII-tubulin: validated for ICC and IF (PubMed IDs: 21965333, 22171041, 24632247, 27229176, 30117103, 31176721)<br>Rabbit anti-GAP43: validated for IHC and IF (PubMed IDs: 21965333, 22171041)<br>Rabbit anti-RBPMS: validated for IHC and IF (PubMed IDs: 31176721, 31784286)<br>Chicken anti-NF-200: validated for IHC and IF (PubMed IDs: 21965333, 27229176, 30117103, 31176721, 32858161)<br>α-Bungarotoxin, Alexa Fluor 488 conjugate: validated for IHC and IF (PubMed IDs: 21965333, 27229176, 30117103, 31176721, 32858161)<br>Rabbit anti-CD68: validated for IHC and IF (PubMed IDs: 33640375, 33947104)

Animals and other organisms

Policy information about studies involving animals; ARRIVE guidelines recommended for reporting animal research

Laboratory animals	Adult male C57BL/6 mice (8-12 weeks old) were used in both in vitro and in vivo experiments.
Wild animals	No wild animals were used in this study.
Field-collected samples	This study did not include samples collected from the field.
Ethics oversight	Animal experiments were performed in accordance with the experimental procedures approved by the Animal Research Ethics Sub-Committee at the City University of Hong Kong.

Note that full information on the approval of the study protocol must also be provided in the manuscript.
